# Revisiting the emerging pathosystem of rice sheath blight: deciphering the *Rhizoctonia solani* virulence, host range, and rice genotype-based resistance

**DOI:** 10.3389/fpls.2024.1499785

**Published:** 2024-12-19

**Authors:** Zeinab A. Kalboush, Yasser S. A. Mazrou, Amr A. Hassan, Ahmed Sherif, Wael E. Gabr, Qurban Ali, Yasser Nehela

**Affiliations:** ^1^ Rice Pathology Department, Plant Pathology Research Institute, Agricultural Research Center, Sakha, Kafrelsheikh, Egypt; ^2^ Business Administration Department, Community College, King Khalid University, Guraiger, Abha, Saudi Arabia; ^3^ Rice Research Department, Field Crops Research Institute, Agricultural Research Center, Sakha, Kafrelsheikh, Egypt; ^4^ Department of Biology, College of Science, United Arab Emirates University, Al-ain, Abu-Dhabi, United Arab Emirates; ^5^ Department of Agricultural Botany, Faculty of Agriculture, Tanta University, Tanta, Egypt

**Keywords:** sheath blight, *rhizoctonia*, host range, rice genotype, SSR marker, weeds, cell wall

## Abstract

Sheath blight, caused by *Rhizoctonia solani* AG1 IA, is a challenging disease of rice worldwide. In the current study, nine *R. solani* isolates, within the anastomosis group AG-1 IA, were isolated, characterized based on their macroscopic and microscopic features, as well as their ability to produce cell wall degrading enzymes (CWDEs), and further molecularly identified via ITS sequencing. Although all isolates were pathogenic and produced typical sheath blight symptoms the susceptible rice cultivar, Sakha 101, *R. solani* AG1 IA -isolate SHBP9 was the most aggressive isolate. The virulence of isolate SHBP9 was correlated with its overproduction of CWDEs, where it had the highest pectinase, amylase, and cellulase activity *in vitro*. *R. solani* AG1 IA -isolate SHBP9 was able to infect 12 common rice-associated weeds from the family Poaceae, as well as over 25 economic crops from different families, except chickpea (*Cicer arietinum*) from Fabaceae, Rocket (*Eruca sativa*) from Brassicaceae, and the four crops from Solanaceae. Additionally, rice genotype-based resistance was evaluated using 11 rice genotypes for their response to *R. solani* isolates, morphological traits, yield components, and using 12 SSR markers linked to sheath blight resistance. Briefly, the tested 11 rice genotypes were divided into three groups; Cluster “I” included only two resistant genotypes (Egyptian Yasmine and Giza 182), Cluster “II” included four moderately resistant genotypes (Egyptian hybrid 1, Giza 178, 181, and 183), whereas Cluster “III” included five susceptible (Sakha 104, 101, 108, Super 300 and Giza 177). Correspondingly, only surface-mycelium growth was microscopically noticed on the resistant cultivar Egyptian Yasmine, as well as the moderately resistant Egyptian hybrid 1, however, on the susceptible Sakha 104, the observed mycelium was branched, shrunk, and formed sclerotia. Accordingly, Indica and Indica/Japonica rice genotypes showed more resistance to *R. solani* than Japonica genotypes. These findings provide insights into its pathogenicity mechanisms and identify potential targets for disease control which ultimately contributes to the development of sustainable eco-friendly disease management strategies. Moreover, our findings might pave the way for developing resistant rice varieties by using more reliable resistance sources of non-host plants, as well as, rice genotype-based resistance as a genetic resource.

## 1 Introduction

Rice (*Oryza sativa* L.; family Poaceae) is a monocotyledonous, semi-aquatic annual cereal crop that is cultivated worldwide across a wide range of land areas and in diverse environmental conditions, from wetlands to uplands and temperate to tropical conditions (Hussain et al., 2020; Nawaz et al., 2022; Zahra et al., 2022). Globally, rice is grown over 165.04 million hectares with a production of 776.46 million tons (FAOSTAT, 2023). In Egypt, rice is cultivated over 646.32 thousand hectares, mainly in the northern and Delta areas, representing only 3.91% of the rice area harvested in Africa (16.52 million hectares). However, this small-harvested area in Egypt produces approximately 5.80 million tons, accounting for 14.54% of the estimated African rice production (39.87 million tons) (FAOSTAT, 2023). This might be due to the high productivity of rice per land unit in Egypt. The Egyptian estimated yield (8.97 tons per hectare) is almost double the global yield per land unit (4.70 tons per hectare) and 3.7-fold of the estimated African yield (2.41 tons per hectare) (FAOSTAT, 2023). Although rice consumption increases annually, the spread of various rice diseases, disorders, and pests is also increasing to pose greater challenges to rice production worldwide. Unfortunately, rice plants are susceptible to numerous fungal, bacterial, viral, and other diseases caused by phytoplasmas and nematodes (Mew et al., 2004; Gnanamanickam and Gnanamanickam, 2009; Cartwright et al., 2018). Fungal diseases of rice include, but are not limited to, rice blast (*Magnaporthe oryzae*; Anamorph: *Pyricularia oryzae*), brown spot (*Bipolaris oryzae*; Anamorph: *Helminthosporium oryzae*), sheath blight (*Rhizoctonia solani*), bakanae disease (*Fusarium fujikuroi*), false smut (*Ustilaginoidea virens*), narrow brown leaf spot (*Cercospora janseana*), kernel smut (*Tilletia barclayana*), and foot rot (*F. moniliforme*) (Mew et al., 2004; Gnanamanickam and Gnanamanickam, 2009; Cartwright et al., 2018).

Among these diseases, Sheath blight (ShB) is a soil-borne disease caused by fungal pathogens (*Rhizoctonia solani)*; it is recorded as one of the most important diseases (Wamishe et al., 2007). The fungus responsible for rice sheath blight disease, *R. solani* Kühn teleomorph *(Thanatephorus cucumeris)* anastomosis group (AG) AG-1 IA, was initially identified in Japan at the start of the early twentieth century (Lee and Rush, 1983), then it has been observed throughout the world’s major rice-growing regions (Ou, 1985). In Egypt, the rice sheath blight was recorded as a serious disease caused by the phytopathogenic fungus *R. solani* AG1 IA (Hassan, 2021). Distinctive mycelium properties of the *Rhizoctonia* fungus as, at the point of branching, typical right angles are usually seen at hyphal constrictions, the mycelium consists of hyphae partitioned into individual cells by dolipore septa, the sclerotia appeared after three days of inoculation on PDA media (Hassan, 2021). The symptoms of the disease include greenish-gray elliptical or oval-shaped spots with yellow margins mostly found on leaf sheaths, but, at times, leaf blades are also infected (Vidhyasekaran et al., 1997).

Plant cell walls are primarily composed of cellulose, hemicellulose, and lignin, with cellulose being the most prevalent component (Han et al., 2003; Keegstra, 2010). Cell wall degradation enzymes (CWDEs) play an important role in the fungus growth by releasing polysaccharides and nutrients, further facilitating hyphal penetration, and increasing branching around the host (De Lorenzo et al., 1997). There is a correlation between the infection severity and activity of CWDEs (Gawade et al., 2017), in the case of *R. solani* IG1 IA isolates, a high ability to produce CWDEs is linked to the rapid appearance of symptoms related to rice sheath blight disease (Hassan, 2021). In the process of fungal infection in plants, CWDEs, particularly pectolytic enzymes, are essential because they break down pectin, which gives the pathogen a carbon source, and expose cell wall components to other enzymes like cellulase and hemicellulase, which further break down the cell wall (Jia et al., 2009). In addition to pectolytic and cellulolytic enzymes, lipases and proteases are key enzymes in pathogenesis that attack the plasmalemma following the breakdown of the cell wall by proteases (Hameed et al., 1994).

*R. solani* has a wide host range and high pathogenicity; the main obstacle in managing sheath blight disease is the lack of an identified germplasm with an adequate level of resistance for use in the resistance breeding program (Poonguzhali et al., 2022), because of the lack of identified resistant donors in cultivated varieties (Bonman et al., 1992) and, until now not determined sources of stable resistance (Molla et al., 2020). About 250 different host plant species, including those of the Poaceae, Fabaceae, Solanaceae, Amaranthaceae, Brassicaceae, Rubiaceae, Malvaceae, Asteraceae, Araceae, Moraceae, and Linaceae families, are susceptible to *R. solani* (Chahal et al., 2003). *R. solani* has a total of 14 distinct anastomosis groups (AG1 to AG13 and AGBI), all of which show significant variation in colony morphology, host range, aggressiveness, and nutritional requirements (Guillemaut et al., 2003; Ahvenniemi et al., 2009; Ajayi-Oyetunde and Bradley, 2018). The three subgroups of *R. solani* AG1 isolates (IA, IB, and IC) are separated based on sclerotia size and morphology as well as sequence homology (Sneh et al., 1991), this all results in rice ShB, with AG1-IA being identified as the causative agent most frequently (Bernardes-De-Assis et al., 2009; Gonzalez et al., 2011). As a result of climatic change, the excessive usage of fungicides to control sheath blight disease is unfriendly to the environment and the general health (Goswami et al., 2019). Moreover, the development of resistant rice genotypes to sheath blight was the most eco-friendly environmental strategy to stop the disease from spreading in the soil (Liu et al., 2009). However, the available breeding strategies are time-consuming (Sherif et al., 2023).

The sheath blight pathogen can produce sclerotia, which may remain dormant over the years in the soil, and stubble and re-infect healthy rice plants in the subsequent rice season (Dey et al., 2016). Long-term and broad-spectrum resistance have been linked to quantitative resistance, which makes it an important tool for rice breeding. Furthermore, the disease resistance is controlled by multiple genes, which confer quantitative resistance (Molla et al., 2020). The revolution of next-generation sequencing and genome-wide association analysis (GWAS) helps in the desiccation of the molecular basis for disease resistance and reduces the breeding cycle for sheath blight resistance (Liu et al., 2021). Recently, two major loci controlling the disease resistance were identified on chromosomes 9 and 11 (Liu et al., 2009; Channamallikarjuna et al., 2010), and then a dozen QTLs were reported on chromosomes 1, 2, 3, 5, 7, and 8 (Chen et al., 2023). Using resistance-linked molecular markers would save time during the breeding programs.

Sheath blight is an emerging disease in Egypt and our knowledge about sources of rice genotypes resistance to sheath blight pathogen is still limited (Hassan, 2021; Kalboush et al., 2024). The current study aims (i) to isolate and characterize the causal agent of sheath blight on rice and to determine the relationship between the pathogen’s virulence and its ability to produce CWDEs which could provide insights into its pathogenicity mechanisms and identify potential targets for disease control. (ii) To determine the host range of the *R. solani* AG1 IA and short-list the non-host plants which could lead to the development of resistant rice varieties using non-host plants as a genetic resource. And (iii) to evaluate the rice genotype-based resistance against *R. solani*, to find more reliable resistance sources that could contribute to the rice breeding programs. The findings of this study could provide a comprehensive understanding of the pathogen, its interaction with its host (s), and the potential for developing resistant rice varieties, ultimately contributing to the development of better sustainable and eco-friendly disease management strategies.

## 2 Materials and methods

### 2.1 Plant materials

Eleven commercial rice cultivars with the pedigree and type; Giza 177 (Giza 171/Yomji No.1/PiNo.4- Japonica), Giza 178 (Giza 175/Milyang 49 - Indica/Japonica), Giza 181 (IR23/IR22- Indica), Egyptian Hybrid 1 (IR69625A/Giza 178 R- Indica/Japonica), Giza 182 (Giza181/IR39422-161-1-3//Giza 181- Indica), Giza 183 (Giza 178/SKC23893- Indica/Japonica), Egyptian Yasmine (IR262/KDML-105- Indica), Sakha 101 (Giza 176/Milyang 79- Japonica), Sakha 104 (GZ 4096-8-1/GZ 4100-9-1- Japonica), Sakha 108 (Sakha 101/HR5824/Sakha 101- Japonica) and Sakha super 300 (PTGMS38/China2- Indica/Japonica) was used as experimental plant materials throughout this study. Seeds were kindly provided by the Rice Research Department, Field Crops Research Institute (FCRI), Agricultural Research Center (ARC), Egypt. Moreover, seeds of 12 common rice-associated weeds (**Table 1**) were obtained from the Weed Research Central Laboratory (WRCL), ARC, Egypt. Likewise, seeds/grains of 31 economic crops (**Table 2**) were generously provided by the Department of Food Legumes Research, FCRI-ARC, Egypt. All seeds were healthy, uniform, and homologous in size and color.

**Table 1:** List of common rice-associated weeds tested for host range assessment of *Rhizoctonia solani* under greenhouse conditions.

No.	Common name	Specific name	Family	Response ^a^
1	Jungle rice	*Echinochloa colona*	Poaceae	+
2	Barnyard grass	*Echinochloa crus-galli*	Poaceae	+
3	Knotgrass	*Paspalum paspalodes*	Poaceae	+
4	Cogongrass	*Imperata cylindrica*	Poaceae	+
5	Wild oat	*Avena fatua*	Poaceae	+
6	Lesser canarygrass	*Phalaris minor*	Poaceae	+
7	Bearded sprangletop	*Leptochloa fusca*	Poaceae	+
8	Hairy finger-grass	*Digitaria sanguinalis*	Poaceae	+
9	Smallflower umbrella-sedge	*Cyperus difformis*	Cyperaceae	+
10	Purple nutsedge	*Cyperus longus*	Cyperaceae	+
11	Foxtail flatsedge	*Cyperus alopecuroides*	Cyperaceae	+
12	Cattail	*Typha elephantina*	Typhaceae	+

a (+) Signify susceptible host, whereas (-) signify non-host.

**Table 2:** List of major economic crops tested for host range assessment of *Rhizoctonia solani* under greenhouse conditions.

No.	Common name	Specific name	Family	Response ^a^
1	Rice	*Oryza sativa*	Poaceae	+
2	Maize	*Zea mays*	Poaceae	+
3	Wheat	*Triticum aestivum*	Poaceae	+
4	Barley	*Hordeum vulgare*	Poaceae	+
5	Sorghum	*Sorghum bicolor*	Poaceae	+
6	Squash	*Cucurbita pepo*	Cucurbitaceae	+
7	Cucumbers	*Cucumis sativus*	Cucurbitaceae	+
8	Watermelon	*Citrullus lanatus*	Cucurbitaceae	+
9	Cotton	*Gossypium herbaceum*	Malvaceae	+
10	Okra	*Abelmoschus esculentus*	Malvaceae	+
11	Flax	*Linum usitatissimum*	Linaceae	+
12	Cowpea	*Vigna unguiculata*	Fabaceae	+
13	Chickpea	*Cicer arietinum*	Fabaceae	–
14	Soybean	*Glycine max*	Fabaceae	+
15	Faba bean	*Vicia faba*	Fabaceae	+
16	Common bean	*Phaseolus vulgaris*	Fabaceae	+
17	Pea	*Pisum sativum*	Fabaceae	+
18	White lupin	*Lupinus albus*	Fabaceae	+
19	Berseem	*Trifolium alexandrinum*	Fabaceae	+
20	Fenugreek	*Trigonella foenum-graecum*	Fabaceae	+
21	Radishes	*Raphanus sativus*	Brassicaceae	+
22	Rocket	*Eruca sativa*	Brassicaceae	–
23	Canola	*Brassica napus* subsp. *napus*	Brassicaceae	+
24	Spinach	*Spinacia oleracea*	Amaranthaceae	+
25	Coriander	*Coriandrum sativum*	Apiaceae	+
26	Dill	*Anethum graveolens*	Apiaceae	+
27	Lettuce	*Lactuca sativa*	Asteraceae	+
28	Potato	*Solanum tuberosum*	Solanaceae	–
29	Tomato	*Solanum lycopersicum*	Solanaceae	–
30	Eggplants	*Solanum melongena*	Solanaceae	–
31	Pepper	*Capsicum annuum*	Solanaceae	–

a (+) Signify susceptible host, whereas (-) signify non-susceptible host.

All experiments were conducted at the Rice Pathology Department, Rice Research and Training Center (RRTC), ARC, Egypt. Seeds were initially sterilized for 3-4 minutes using sodium hypochlorite (NaClO) solution (1%) and then washed twice with sterile water, then planted in plastic pots (30 cm inner diameter and 45 cm in depth) filled with sterilized clay soils. All pots were maintained under greenhouse conditions at 27 ± 2°C, >75% RH, and 8D:16L light cycle. Plants were irrigated daily, or earlier if needed, and fertilized with macro- and micronutrients according to the recommendations of the Egyptian Ministry of Agriculture and Land Reclamation for these cultivars/species.

### 2.2 Isolation, characterization, and molecular identification of *R. solani* isolates

#### 2.2.1 Pathogen isolation

Samples of rice plants showing typical symptoms of sheath blight disease (disease severity: 20-40%) were collected from commercial farms within three different governorates (Beheira, Dakahlia, and Kafrelsheikh) (**Table 3**). Initially, collected infected materials were surface sterilized for 3-4 min using 1% NaOCl, then washed twice using sterilized distilled water, and dried between sterilized filter papers to remove the extra water. The fungal causal agents were isolated as described by Hassan et al. (2023). Briefly, the sterilized parts were placed on a water agar medium (WA), and then incubated at 26 ± 2°C for 24 h. moreover, the hyphal tips method (Brown, 1924) was used to purify the fungal isolates from the mixed or contaminated cultures. Briefly, the terminal of the growing hypha was cut off and transferred to a new potato dextrose agar (PDA) then incubated at 26 ± 2°C until the whole surface of the plate was covered with mycelium.

**Table 3:** Sources and plant materials used to isolate the causal agent of rice sheath blight disease used in this study.

Isolate No.	Governate	City	Rice cultivar
SHBP1	Beheira	Itaielbarood	Sakha super 300
SHBP2	Beheira	Abohomos	Sakha 108
SHBP3	Beheira	Elebrahimyia	Giza 178
SHBP4	Dakahlia	Dekerns	Sakha 108
SHBP5	Dakahlia	Talkha	Sakha 104
SHBP6	Dakahlia	Mansoura	Sakha 108
SHBP7	Kafrelsheikh	Sakha	Sakha super 300
SHBP8	Kafrelsheikh	Misaar	Sakha 108
SHBP9	Kafrelsheikh	Misaar	Sakha super 300

#### 2.2.2 Macroscopic and microscopic characterization of *R. solani* isolates

All fungal isolates were initially identified based on their macroscopic features (cultural morphology), and microscopic characteristics (mycelium morphology, and other observed structures) (Barnett and Hunter, 1972). Briefly, 9-cm Petri dishes containing PDA media were inoculated at the center with a 0.5-cm plug of three-day-old cultures of the fungal isolates and incubated for seven days as described above. All tested isolates were examined for morphological characteristics according to Moni et al. (2016). Each isolate was maintained with five biological replicates and repeated twice (two technical replicates). The purified fungal isolates were first identified based on their cultural morphology characteristics and then the identification was confirmed using microscopic features as *R. solani*.

#### 2.2.3 Molecular identification of *R. solani* isolates

Moreover, the identification of the nine isolates was further confirmed based on the sequencing of the Internal transcribed spacer (ITS) region. Briefly, the isolates were grown on PD broth and incubated at 26 ± 2°C for 5-7 days. Then, the fungal mycelium was harvested, filtered through cheesecloth, washed twice with sterile water, and dried using sterile filter paper. The genomic DNA was extracted according to Kuramae (1997) with slight modifications as described by Atallah et al. (2022, 2024). Subsequently, the 18S rDNA fragment was amplified using the ITS1 (5’ TCCGTAGGTGAACCTGCGG 3’) and ITS4 (5’ CCTCCGCTTATTGATATGC 3’) universal primers (Usyk et al., 2017). Molecular analysis was done through the Sigma Company, Germany. The query sequence was compared to the recent available data in Gen Bank, the National Center for Biotechnology Information website (NCBI, http://www.ncbi.nlm.nih.gov/gene/; access) using the Nucleotide–Nucleotide Basic Local Alignment Search Tool (BLASTn) (Altschul et al., 1997). Due to the variation in the length of ITS-based sequence accessions, sequences were initially aligned using the ClustalW algorism, then trimmed (~480 bp) while used for phylogenetic analysis. The Molecular Evolutionary Genetics Analysis software (MEGA11) was used to align the query sequence with other 40 *Rhizoctonia* strains/isolates from different anastomosis groups (AGs) retrieved from the recently available data in NCBI GenBank (**Table 4**). The Neighbor-Joining method was used to infer the phylogenetic tree for the identified isolates (Saitou and Nei, 1987). Furthermore, the phylogeny test was done using the Bootstrap method.

**Table 4:** Sequences of internal transcribed spacer (ITS) region from *Rhizoctonia* strains/isolates from different anastomosis groups (AGs) used for phylogenetic analysis.

Anastomosis group	Isolate/culture collection	GenBank	Accession length (bp)	Host	Origin
AG-1 IA	F190	FJ492099.3	644	Sugar beet	Japan
AG-1 IA	SHBP1	PP445281.1	483	Rice	Egypt
AG-1 IA	SHBP2	PP426068.1	612	Rice	Egypt
AG-1 IA	SHBP3	PP426196.1	681	Rice	Egypt
AG-1 IA	SHBP4	PP426588.1	681	Rice	Egypt
AG-1 IA	SHBP5	PP445282.1	483	Rice	Egypt
AG-1 IA	SHBP6	PP426616.1	686	Rice	Egypt
AG-1 IA	SHBP7	PP445324.1	483	Rice	Egypt
AG-1 IA	SHBP8	PP429228.1	481	Rice	Egypt
AG-1 IA	SHBP9	PP445325.1	681	Rice	Egypt
AG-1 IB	Rs-16	MT568768.1	692	*Brassica oleracea*	Turkey
AG-1 IC	F192	FJ492101.3	636	Sugar beet	Japan
AG-2-1	F193	FJ492102.3	654	Sugar beet	Japan
AG-2-2	UP105	MH465659.1	699	Rice	India
AG-2-2 IIIB	F561	FJ492171.3	684	*Phaseolus vulgaris*	North Dakota, USA
AG-2-2 IV	F24	FJ492083.3	685	Sugar beet	Idaho, USA
AG 2-3	–	HM054532.1	401	Chickpea	Tunisia
AG-3	voucher EGY-RS1023	PQ200681.1	668	Potato	Egypt
AG-3 PT	SX-2H	KJ170333.1	703	Wheat	Turkey
AG-3 TA21	–	KX852461.1	680	Tobacco	China
AG-4	–	JF792353.1	694	Tobacco	Argentina
AG4-3	–	OQ794049.1	634	Watermelon	USA
AG-4 HG	10Bu19	OL762321.1	655	*Phaseolus vulgaris*	Turkey
AG-4 HG I	Rz14-7	MF474259.1	484	Cilantro	California, USA
AG-4 HG II	ND12	HQ629872.1	678	*Pisum sativum*	North Dakota, USA
AG-4 HG III	IBRS02	KF746163.1	721	Gypsophila	Sao Paulo State, Brazil
AG-5	strain RIZ-46F	MK852270.1	624	Potato	France
AG-5	DB132	OM039417.1	691	*Phaseolus vulgaris*	USA
AG-6	AG-6_Carling	KX118332.1	671	Soybean	Illinois, USA
AG-7	HNDA02-1	KF907734.1	676	*Brassica oleracea*	Vietnam
AG-8	4254	KC590555.1	716	Wheat	Turkey
AG-8	F201	FJ492109.3	613	Sugar beet	Australia
AG-10	Rh 120203	DQ356410.1	677	Spring wheat	USA: Washington, Pullman
AG-10	Rh 100278	DQ356408.1	714	Chickpea	USA: Washington, Walla Walla
AG-11	DB63	OM045069.1	696	*Phaseolus vulgaris*	USA
AG-11	P174	MT177257.1	611	*Pisum sativum*	Canada
AG-A	336_29	MT530327.1	613	Sugar beet	Turkey
AG-A	U133	MT177258.1	592	*Lactuca sativa*	Canada
AG-BI	523-1	MT177260.1	664	*Lactuca sativa*	Canada
AG-E	534-6	MT177262.1	624	*Triticum* sp.	Canada
AG-F	F14	OL762325.1	617	*Phaseolus vulgaris*	Turkey
AG-Fa	SPM1	KX674533.1	715	Spinach	Malaysia
AG-Fc	LDDL02-1	KF907736.1	678	*Brassica chinensis*	Vietnam
AG-G	strain Rh286	OL405126.1	593	*Olea europaea*	Italy
AG-H	534-2	MT177264.1	458	*Triticum* sp.	Canada
AG-I	390.Rs.16-70	MT177265.1	665	*Glycine max*	Canada
AG-K	F523	FJ492158.3	625	Sugar beet	Idaho, USA
AG-K	78_352_AGK_RHIZ	MT476623.1	620	Sugar beet	Turkey
AG-R	strain Di3 ARM4	MG263522.1	613	*Mandevilla sanderi*	Italy

The listed accessions were retrieved from the most recent available data in GenBank, the National Center for Biotechnology Information website (NCBI, http://www.ncbi.nlm.nih.gov/gene/).

**Figure 1:**
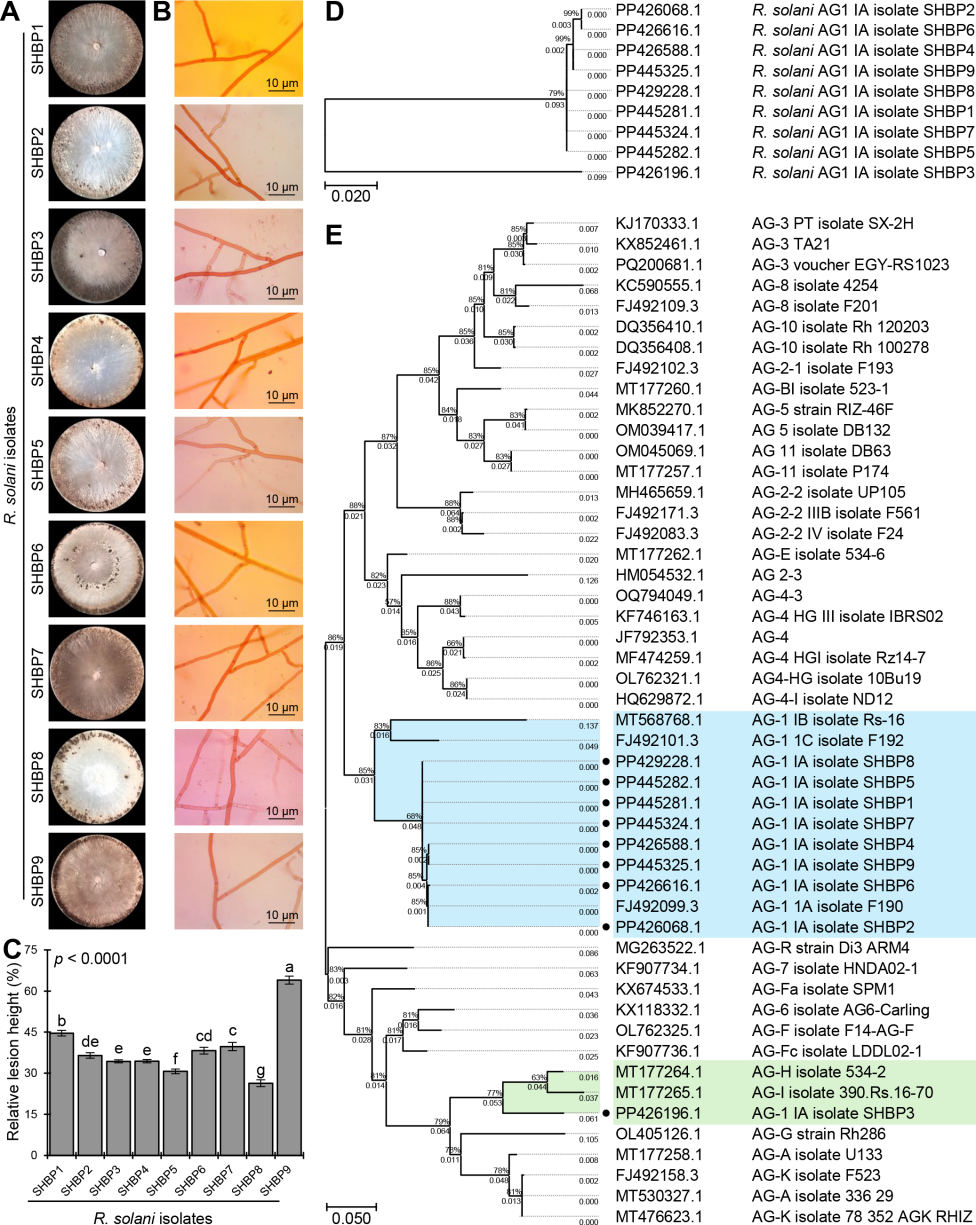
The macroscopic and microscopic features, pathogenicity, and molecular identification of the phytopathogenic fungus *Rhizoctonia solani*, the causal agent of sheath blight disease on rice. **(A)** The growth and macroscopic features of the nine isolates of *R. solani* on potato dextrose agar (PDA) media after 10 days of incubation at 26 ± 2°C. **(B)** Microscopic characteristics and mycelium features of the nine isolates of *R. solani*. **(C)** The relative lesion height (%) of nine isolates of *R. solani* on susceptible rice cultivar Sakha 101 under greenhouse conditions. Bars denote the means ± standard deviations (means ± SD) of five biological replicates. Different letters signify statistically significant differences among isolates using the Tukey HSD test (p< 0.05). **(D, E)** The evolutionary analysis of only *R. solani* AG1 IA isolates SHBP 1-9 by themselves and in comparison with other 40 *R. solani* strains/isolates from different anastomosis groups (AGs) retrieved from the recent available data in National Center for Biotechnology Information (NCBI) GenBank (https://www.ncbi.nlm.nih.gov/). The evolutionary history was inferred using the Maximum Likelihood method and the Tamura-Nei model. The tree with the highest log likelihood ( (-1242.36 and -6738.22, respectively) is shown. The percentage of trees in which the associated taxa clustered together is shown next to the branches. The proportion of sites where at least 1 unambiguous base is present in at least 1 sequence for each descendent clade is shown next to each internal node in the tree. Analysis in panel D involved 9 nucleotide sequences with a total of 608 positions in the final dataset, whereas analysis in panel E involved 49 nucleotide sequences with a total of 694 positions in the final dataset. There were Evolutionary analyses were conducted in MEGA11.

Listed fungal genera were used to generate the phylogenetic tree presented in **Figures 1D, E**.

#### 2.2.4 Pathogenicity of *R. solani* isolates

To fulfill Koch’s postulates, the pathogenicity of the nine *R. solani* isolates was assessed on the commercial susceptible rice cultivar ‘Sakha 101’ as described by Park et al. (2008) with slight modifications. Briefly, rice seeds were sterilized for 3-4 minutes using sodium hypochlorite (NaClO) solution (1%) and then washed twice with sterile water. The sterilized seeds were planted in sterile soil until the seedlings reached about twenty-one days of age. Afterward, the seedlings were transferred to new sterilized soil as individual plants in each pot (30 cm in diameter) had two seedlings. The seedlings were maintained and fertilized well (1 g 46.5% N/pot) until they reached 55 days from planting and produced the maximum number of branches. The isolates were prepared and inoculated on the sheaths of rice plants using a 5-mm plug of three-day-old cultures of each isolate separately. Fungal plugs were placed on the sheaths, starting from the base of the plants, and the pieces were wrapped in aluminum foil. After 3-4 days of inoculation, the aluminum foil was removed. Plants were monitored at 30 ± 2°C with a relative humidity of 80-100% until symptoms appeared. This experiment was completely randomized with five replicates, each with four pots. The experiment was repeated twice under the same conditions. Disease scoring was calculated as described by the International Rice Research Institute’s standard evaluation system (IRRI, 2013) on a scale of 0 to 9. The disease severity was taken after one month of symptoms appeared, as relative lesion height (RLH) using the formula described by Sharma and Teng (1990).

#### 2.2.5 *in vitro* enzymatic activity of cell wall degradation enzymes of *R. solani* isolates

The screening of cell wall degradation enzymes (CWDE) including cellulase, pectinase, and amylase activity was assessed by transferring a 5-mm mycelial plug of a 7-day-old culture of each *R. solani* isolate on the media containing carboxymethyl cellulose (CMC), pectin, or starch, respectively (Leopold and SamsˇinˇÁková, 1970; Hankin and Anagnostakis, 1975; Jalgaonwala and Mahajan, 2011), then incubated at 26 ± 2°C for 3-7 days. To visualize the CWDE activities, CMC and pectin media with the *R. solani* were flooded with 0.3% Congo red solution for 15min and plates were destained with 0.1% NaCl for another 15 minutes, whereas the plates with starch agar medium were flooded with iodine solution, kept for one minute, and then poured off. The plates were visualized for the halo/clear zone, indicating cellulase, pectinase, and amylase production (Anand et al., 2010). Data were expressed as Fungal linear growth (cm) as an indicator for better utilization of CMC, pectin, or starch from the media.

#### 2.2.6 Expression of cell wall degradation genes of *R. solani* isolates using real-time PCR

Fungal mycelia of *R. solani* were collected after 3 days of incubation on the media containing pectin to assess the gene expression of some CWD genes including pectate lyase B (*RsPLB*; AG1IA_00690) with sequencing F (GTCGGGACGGTAAGCATAAA) and R (GCTAGCTTCAGAGGCGAATAA), putative pectate lyase C (*RsPLC*; AG1IA_10120) with sequencing F (TATTCGACCATGTCAGCGTATC) and R (GAATGTCTCATCTGCCTTCCA), and polysaccharide lyase family 1 protein (*RsPSL*; AG1IA_06890) with sequencing F (AGGACTACTACGATGGGTTACT) and R (TCTTCGTCTTCGTTGCTATCC), using qRT-PCR. Briefly, total fungal RNA was isolated using a Simply P Total RNA Extraction Kit (catalog number BSC52S1), according to the manufacturer’s procedure. Then, cDNA was synthesized using a TOP script™ cDNA Synthesis Kit as described in the manufacturer’s protocol. The primer sequences for the three genes (*RsPLB*, *RsPLC*, and *RsPSL*) are previously described by (Bhaskar Rao et al., 2020). *β*-Tubulin was used as a housekeeping gene, and the 2^−ΔΔCT^ method was used for the calculation of relative gene expression (Livak and Schmittgen, 2001). The expression level of these genes in *R. solani* grown on a pectin-free medium was used as a reference point for comparison.

### 2.3 Greenhouse experiments

#### 2.3.1 Host range assessment of *R. solani* AG1 IA isolate SHBP9 under greenhouse conditions

Seeds of 12 common rice-associated weeds (**Table 1**), and 31 economic crops (**Table 2**) were initially sterilized using NaClO (1%) for 3-4 min, washed twice with sterile water, and then transferred into sterilized conical flasks. A 5-mm mycelial plug of a 7-day-old culture from *R. solani* AG1 IA isolate SHBP9 was transferred separately into each flask with a mixture of sterilized corn, rice husk, and sand (1:2:1). The flasks were incubated at 26 ± 2°C for 15 days until the fungus produced the sclerotia. The pots (30 cm) containing sterile soil were inoculated with sandy media containing *R. solani* AG1 IA isolate SHBP9 and mixed well, then irrigated and maintained under greenhouse conditions for about 24h. The seeds were sown in the inoculated pots, covered with sterile soil, irrigated again, then maintained at 26 ± 2°C under greenhouse conditions. After 21-28 days of sowing for different crops, the disease symptoms were developed and recorded as (+) susceptible host and (-) for non-host. On the other hand, weed plants were left to 60 days post-sowing (dps), and the disease score was recorded. A complete randomized design with three replicates was used for the experiment; each replicate had four pots. The whole experiment was repeated twice to ensure the reliability and validity of the results.

#### 2.3.2 Evaluation of rice genotypes under artificial inoculation with *R. solani* AG1 IA (SHBP9 isolate) at greenhouse conditions

Eleven rice genotypes were used in this experiment. The seeds of rice genotypes were initially sterilized and sown as described above. Subsequently, rice seedlings were thinned as 2 seedlings per pot and fertilized as recommended (1 g 46.5% N/pot) at 21 dps and maintained under greenhouse conditions as described above. At 60 dps, rice plants were inoculated with *R. solani* AG1 IA isolate SHBP9 as described by Park et al. (2008). The inoculated genotypes were monitored at 26 ± 2°C with a relative humidity of 80-100% until symptoms appeared. The plants were observed for symptom expression after 30 days post-inoculation (dpi). The rice genotypes inoculated with PDA plugs without any fungal mycelia served as negative control (Mock) plants and were used for comparison of the vegetative and yield parameters in *R. solani-*inoculated and non-inoculated (Mock) rice genotypes. The whole experiment was repeated twice to ensure the reliability and validity of the results.

##### 2.3.2.1 Assessment of sheath blight disease

Disease scoring for rice genotypes was calculated as described by IRRI (2013) on a scale of 0 to 9. The disease severity was taken after 30 days of symptoms appearance, as Relative lesion height (RLH) and the Percent disease index (PDI) were calculated using the formula provided by Sharma and Teng (1990) and Naveenkumar et al. (2022), respectively.


$$RLH=\left(\frac{Lesion\;height}{Plant\;height}\right)\times \;100$$

$$PDI=\left(\frac{Sum\;of\;all\;ratings}{Total\;number\;of\;observations\;\times \;Maximum\;rating\;scale}\right)\times \;100$$

##### 2.3.2.2 Assessment of vegetative and yield parameters

The vegetative traits including plant height (cm) at the maturing stage, number of tillers per plant, and stem diameter (mm) at the maximum tiller stage were recorded. Moreover, flag leaf area (FLA; cm^2^) was calculated using the formula FLA = K (L × W) of Palaniswamy and Gomez (1974), where L is the maximum leaf length, W is the maximum width of the leaf and K=0.75. Likewise, the yield components including panicle length (cm), panicle weight (g),1000-grain weight (g), and the number of discolored grains were recorded for *R. solani*-infected and non-infected rice genotype plants at the harvest stage. All parameters were collected according to the guidelines described in IRRI (2002).

##### 2.3.2.3 Preparation of rice sheath for microscopy

Infected materials were collected from the tested eleven rice genotypes at 7 dpi. Subsequently, each infected sheath was chopped into small pieces (2-5 mm), submerged in trypan blue solution (0.05%) for 10 min, then washed with lactophenol three times. After washing, stained samples were transferred onto glass slides and examined using a compound microscope (Basu et al., 2016).

#### 2.3.3 Molecular analysis using simple sequence repeat markers

Young leaves of eleven rice genotypes were collected for DNA extraction according to Murray and Thompson (1980). Furthermore, the purity of the extracted DNA was checked by Agarose gel (0.8%) and stored at -20°C for further investigations. Twelve SSR markers (**Table 5**) were selected from the previous studies on sheath blight. The polymerase chain reaction was conducted in 20 µl reaction volume containing 2 µl DNA templet (10 ng), 10 ul master mix (2X Taq Plus Master Mix with dye), 0.5 ul of each forward and reversed primers (10 pM), and 7 µl of d_2_H_2_O. In addition, the gel electrophoresis was done using Agarose gel of 3%, and the band size was calculated by GelAnalyzer 23.1.1 (www.gelanalyzer.com). The cluster analysis and its associated dendrogram were prepared by Paleontological Statistics (Past 4.13) software (Hammer and Harper, 2001).

**Table 5:** Primers used for simple sequence repeat (SSR) markers in this study.

Marker names	Chr	Direction	Primer sequence (5'-3')	Product Size (bp)	Annealing temperature (°C)	Reference
RM202	11	F R	TGGAACACCCATAGACAAACAGC TGGCAAGTGGTATTCTTCCTTCC	291	50	(Chen et al., 1997)
RM224	11	F R	ATCGATCGATCTTCACGAGG TGCTATAAAAGGCATTCGGG	123-157	55	(Chen et al., 1997)
RM426	3	F R	ATGAGATGAGTTCAAGGCCC AACTCTGTACCTCCATCGCC	150	55	(Temnykh et al., 2001)
RM6971	9	F R	TTTGCGAACTAGACAAGGCC GCGTCATTCTCGACGAGC	202	55	(McCouch et al., 2002)
RM16	3	F R	CGCTAGGGCAGCATCTAAA AACACAGCAGGTACGCGC	181	55	(Panaud et al., 1996)
RM279	2	F R	GCGGGAGAGGGATCTCCT GGCTAGGAGTTAACCTCGCG	164-174	55	(Temnykh et al., 2000)
RM518	4	F R	CTCTTCACTCACTCACCATGG ATCCATCTGGAGCAAGCAAC	171	55	(Temnykh et al., 2001)
RM570	3	F R	GTTCTTCAACTCCCAGTGCG TGACGATGTGGAAGAGCAAG	208	55	(Temnykh et al., 2001)
RM178	5	F R	TCGCGTGAAAGATAAGCGGCGC GATCACCGTTCCCTCCGCCTGC	117	55	(Temnykh et al., 2000)
RM251	3	F R	ATGCGGTTCAAGATTCGATC ATGCGGTTCAAGATTCGATC	147-118	55	(Chen et al., 1997)
RM335	4	F R	GTACACACCCACATCGAGAAG GCTCTATGCGAGTATCCATGG	104-114	55	(Temnykh et al., 2000)
RM257	9	F R	CAGTTCCGAGCAAGAGTACTC GGATCGGACGTGGCATATG	147-164	55	(Chen et al., 1997)

### 2.4 Data analysis

All *in vitro* experiments were performed in a completely randomized design with five biological replicates and two technical replicates. Likewise, all *in vivo* experiments were performed in a completely randomized design, unless otherwise stated, with three replicates, each replicate had four pots, and all experiment was repeated twice. All data were statistically analyzed according to the analysis of variance technique (ANOVA), followed by Tukey’s Honestly Significant Difference (HSD) Test as a *post hoc* analysis. On the other hand, the experiment of evaluation of rice genotypes against *R. solani* infection was laid out using a split-plot design comprising *R. solani* infection in the main plots (infection) and eleven rice genotypes in subplots (genotypes). Data were statistically analyzed using ANOVA, followed by the HSD Test as a *post hoc* analysis based on the *p*-value of infection (*p*_infection_< 0.05), genotypes (*p*_genotypes_< 0.05), and their interaction (*p*_infection × genotypes_< 0.05). Principal component analysis (PCA) was performed and its associated scatter and loading plots were generated using the response of the 11 tested rice genotypes to the 9 isolates of *R. solani* as expressed by relative lesion height (RLH; %). Moreover, two-way Hierarchical Cluster Analysis (HCA) was performed using the average of each RLH in each treatment. Distance and linkage were done using the Ward method (Ward Jr, 1963) and RLH similarities were presented as a heat map.

## 3 Results

### 3.1 Isolation and Identification of *R. solani* isolates

Nine isolates (named SHBP 1- 9) were isolated from diseased rice cultivars showing typical symptoms of sheath blight disease (four isolates from Sakha 108, three isolates from Sakha super 300, and one isolate from Giza 178 and Sakha 104 each) collected from three Egyptian governorates included Beheira (Itaielbarood, Abohomos, Elebrahimyia), Dakahlia (Dekerns, Talkha, Mansoura), and Kafrelsheikh (Sakha, Misaar, Kellan) (**Table 3**). Isolates SHBP 1-9 showed typical morphological characteristics, colony texture, and abundance of asexual reproductive structures (mycelium and sclerotia) with *R. solani* when grown on a PDA medium (**Figure 1A**). The mycelium growth showed two types: moderate speed with flat shape in five isolates (SHBP1, 3, 4, 7, and 9) and rapid with flat/arial in four isolates (SHBP2, 5, 6, and 8) (**Figure 1A**; **Table 6**).

**Table 6:** Morphological characterization of *Rhizoctonia solani* isolates, the causal fungus of rice sheath blight disease ^a^.

Isolate	Mycelial growth			Sclerotia		GenBank Accession No.	Accession length (bp)
	MGN ^b^	Color	MGS ^c^	NDS ^d^	Shape		
SHBP1	Flat	Dark brown	Moderate	5	Peripheral	PP445281.1	483
SHBP2	Flat/aerial	Cream	Rapid	5	Peripheral	PP426068.1	612
SHBP3	Flat	Dark brown	Moderate	5	Peripheral	PP426196.1	681
SHBP4	Flat	Light brown	Moderate	9	Peripheral	PP426588.1	681
SHBP5	Flat/aerial	Light brown	Rapid	5	Scattered	PP445282.1	483
SHBP6	Flat/aerial	Light brown	Rapid	5	Central/Peripheral	PP426616.1	686
SHBP7	Flat	Dark brown	Moderate	5	Scattered	PP445324.1	483
SHBP8	Flat/aerial	Cream	Rapid	7	Scattered	PP429228.1	481
SHBP9	Flat	Dark brown	Moderate	9	Scattered	PP445325.1	681

a All observations were recorded 15 days after incubation using 5 replicates.

b MGN, mycelium growth nature.

c MGS, Mycelium growth speed; Moderate, the fungal mycelium covers the Petri plate (90mm diameter) in 72 h, whereas Rapid: the fungal mycelium covers the Petri plate (90mm diameter) in 48 h.

d NDS, number of days to produce sclerotia.

Furthermore, we noticed four color patterns: cream (two isolates), light, and dark brown (three isolates each), and a unique isolate, SHBP3, with a black-brown color (**Figure 1A**). Furthermore, the sclerotia appeared in two shapes: peripheral in four isolates (SHBP1, 2, 3, and 4) and scattered in four isolates (SHBP5, 7, 8, and 9); meanwhile, SHBP6 showed a central peripheral shape. In addition, SHBP 1, 2, 5, 6, and 7 recorded the least number of days to produce sclerotia (5 days), and SHBP4 and 9 recorded the greatest number of days to produce sclerotia (9 days) (**Figure 1A** and **Table 6**).

*In situ* microscopic observation revealed that the mycelium is generally dark or hyaline, its cells are lengthy, and its branch septa typically branch off from the main hyphae. Normal right angles were typically seen at the hyphal constrictions at the point of branching (**Figure 1B**). The mycelium was made up of hyphae that have been divided into separate cells by dolipore septa, which have extensive taxonomic characteristics.

### 3.2 *R. solani* AG1 IA - isolate SHBP9 is the most aggressive isolate

Although the nine isolates were pathogenic and produced typical symptoms of sheath blight disease on the susceptible rice cultivar (Sakha 101) under greenhouse conditions, SHBP 9 was the most aggressive one and resulted in the highest relative lesion height % (63.98± 1.45%) on infected rice plants (**Figure 1C**). On the other hand, SHBP8 showed the lowest performance (26.36± 1.23%) compared with all the isolates.

### 3.3 Molecular identification of *R. solani* isolates

The identification of the nine *R. solani* isolates (SHBP1 to 9) was further confirmed based on the sequencing of the Internal transcribed spacer (ITS) region. Briefly, nine PCR products ranged from 481 to 686 bp. The ITS sequence of each isolate was aligned on NCBI for molecular identification, and then each sequence was submitted to the GenBank sequence database of the National Center for Biotechnology Information (NCBI; https://www.ncbi.nlm.nih.gov/). Finally, the isolates accession numbers were as fellow; PP445281, PP426068, PP426196, PP426588, PP445282, PP426616, PP445324, PP429228 and PP445325, for SHBP1 to 9 respectively. The phylogenetic analysis between the nine *R. solani* isolates showed high similarity (about 99%) between isolates except *R. solani* AG1 IA isolate SHBP3 (PP426196.1; 79%) which was clustered separately at the bottom of the dendrogram (**Figure 1D**). it is worth noting that the most aggressive isolate SHBP9 (PP445325.1) was more similar to *R. solani* AG1 IA isolate SHBP4 (PP426588.1) than other isolates.

Moreover, to better understand the relationship between the *R. solani* AG1 IA and other anastomosis groups (AGs), the evolutionary analysis of *R. solani* AG1 IA (isolates SHBP 1-9) in comparison with other 40 *R. solani* strains/isolates from different anastomosis groups (AGs) (**Figure 1E**). Interestingly, eight of the nine *R. solani* isolates (except SHBP3) showed high similarity with the Japanese isolate of *R. solani* AG-1 1A isolate F190 (GenBank accession No. FJ492099.3, 644 bp) isolated from sugar beet. Moreover, the same eight isolates were also clustered with the Turkish isolate AG-1 IB isolate Rs-16 (GenBank accession No. MT568768.1, 692 bp) isolated from Brassica oleracea and the Japanese one AG-1 1C isolate F192 (GenBank accession No. FJ492101.3, 636 bp) isolated from sugar beet (**Figure 1E**). On the other hand, *R. solani* AG1 IA isolate SHBP3 was clustered separately from other isolates and shared similar evolutionary behavior with two Canadian isolates including *R. solani* AG-H isolate 534-2 (GenBank accession No. MT177264.1; 458 bp) isolated from *Triticum* sp. and *R. solani* AG-I isolate 390.Rs.16-70 (GenBank accession No. MT177265.1; 665 bp) from *Glycine max* (**Figure 1E**).

### 3.4 *R. solani* isolates differentially produce cell wall degradation enzymes *in vitro*

**Figure 2:**
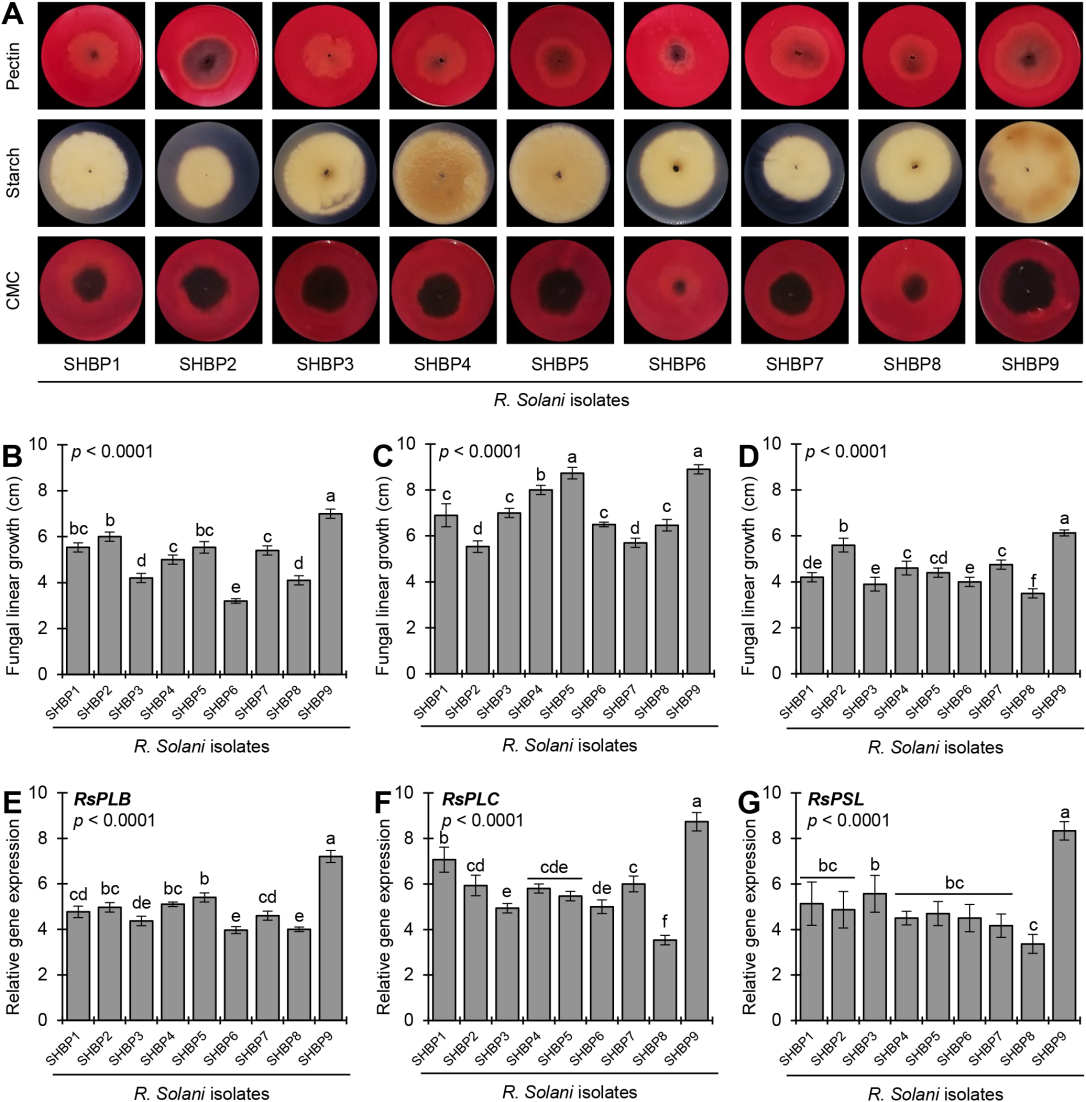
*In vitro* production of cell wall degradation enzymes (CWDEs) by the phytopathogenic fungus *Rhizoctonia solani*, the causal agent of sheath blight disease on rice. **(A)** *In-situ* visualization of CWDEs production of the nine isolates of *R. solani* growing on a minimal inductive medium supplemented with plant-derived polysaccharides (e.g., pectin, starch, or carboxymethyl cellulose [CMC]) for 7 days at 26 ± 2°C. Congo red solution (0.3%) was used to visualize the degradation of pectin and CMC as a halo zone, whereas iodine solution was used for the degradation of starch. **(B-D)** Fungal linear growth (cm) of nine isolates of *R. solani* growing on a minimal inductive medium supplemented with pectin, starch, or CMC, respectively. **(E-G)** Relative gene expression of pectate lyase B (*RsPLB*), putative pectate lyase C (*RsPLC*), and polysaccharide lyase family 1 protein (*RsPSL*), respectively, of nine isolates of *R. solani* growing on inductive pectin-containing medium for 4 days at 26 ± 2°C. Bars represent the average of five biological replicates (n = 5), whereas whiskers represent the standard deviation (means ± SD). Different letters signify statistically significant differences among isolates using the Tukey HSD test (*p*< 0.05).

The nine isolates showed a wide response for the cell wall degradation enzymes (CWDE) production when cultured on a minimal inductive medium supplemented with plant-derived polysaccharides (e.g., pectin, starch, or carboxymethyl cellulose [CMC]) (**Figure 2A**). It is well known that fungal linear growth rates on CWDE-inductive media are correlated with enzyme production. Faster growth on inductive media indicates robust CWDE production. The fungal linear growth of *R. solani* isolates on pectin-containing medium ranged from 3.2 to 7.0 cm (**Figure 2B**), whereas it ranged from 5.5 to 8.9 cm on starch-containing medium (**Figure 2C**) and ranged from 3.9-6.13 cm on CMC-containing medium (**Figure 2D**). *R. solani* AG1 IA isolate SHBP9 recorded the highest linear growth on pectin-, starch-, and CMC- containing media (7.00 ± 0.20, 8.90 ± 0.20, and 6.13 ± 0.13 cm, respectively).

Likewise, the gene expression of pectate lyase B (*RsPLB*, **Figure 2E**), putative pectate lyase C (*RsPLC*, **Figure 2F**), and polysaccharide lyase family 1 protein (*RsPSL*, **Figure 2G**) supported the enzymatic activity analysis. The studied genes were upregulated in all *R. solani* isolates when grown on a pectin-containing medium compared with those grown on a pectin-free medium. For instance, *R. solani* AG1 IA isolate SHBP9 had the highest transcript levels of *RsPLB* (up to 7.20-folds), *RsPLC* (8.73-folds), and *RsPSL* (8.33-folds). On the other hand, *R. solani* AG1 IA isolate SHBP8 had the lowest transcript levels of *RsPLB*, *RsPLC*, and *RsPSL* (4.00-, 3.53-, and 3.37-folds, respectively) when grown on pectin-containing medium compared with those grown on pectin-free medium (**Figures 2E–G**).

### 3.5 Pathogenicity of *R. solani* isolates on different Egyptian rice genotypes

**Figure 3:**
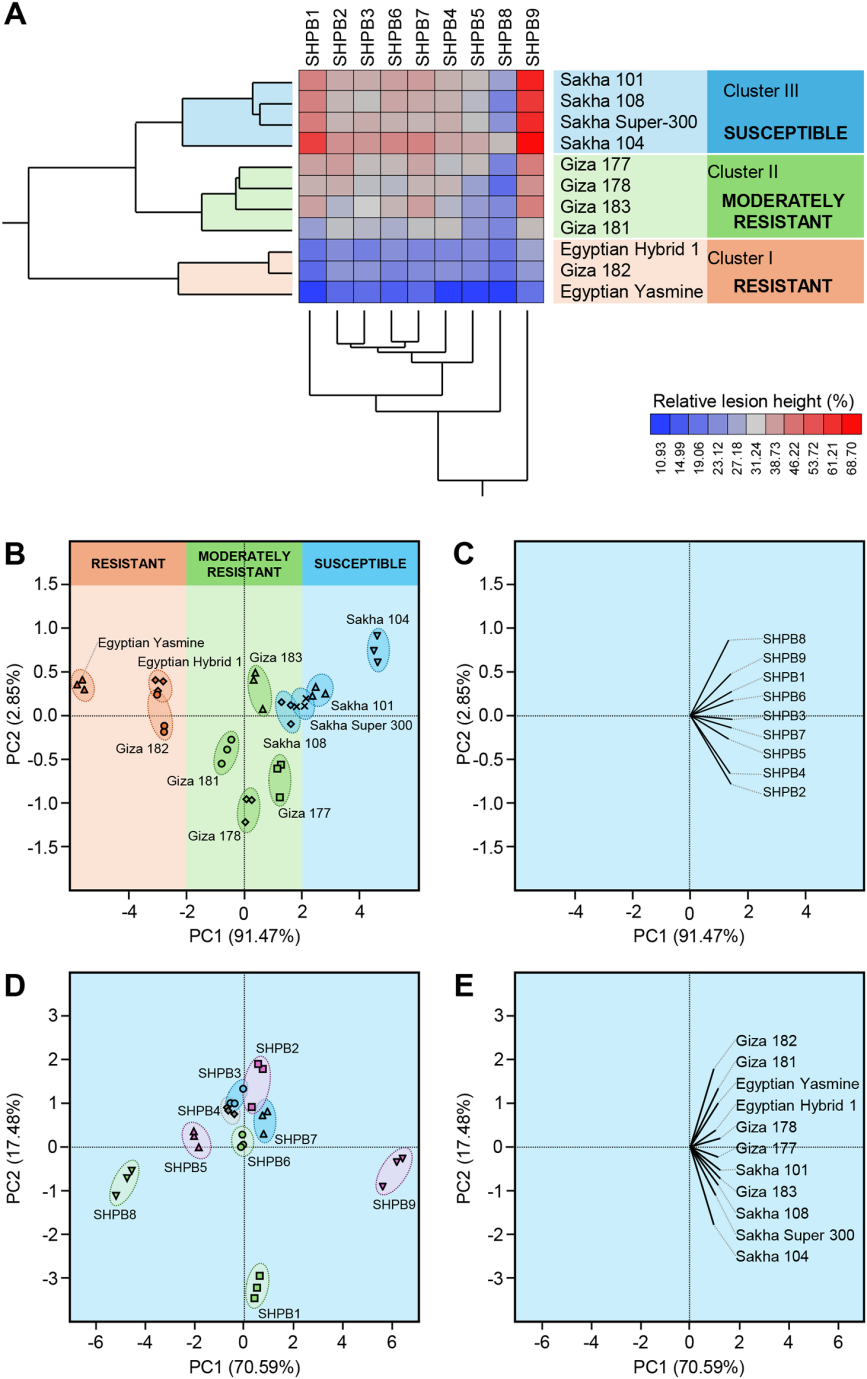
Two-way hierarchical cluster analysis (HCA) and Principal component analysis (PCA) of relative lesion height (%) of 11 commercial rice cultivars after the infection with individual *Rhizoctonia solani* AG1 IA Isolates (SHBP 1-9). **(A)** HCA-associated eat map and cluster dendrograms of relative lesion height (%) of 11 commercial rice cultivars after the infection with individual *R. solani* AG1 IA Isolates (SHBP 1-9). The differences in relative lesion height (%) are visualized in the heat map diagram. Isolates are presented in columns and rice cultivars are presented in rows. Isolates and cultivars are organized using two-way hierarchical cluster analysis based on similarities in auto-scaled values and correlations, respectively. Low relative lesion height (%) is colored blue, whereas higher relative lesion height (%) is colored red (see the scale at the right bottom corner of the graph). **(B, D)** PCA-associated scatter plots of rice cultivars and *R. solani* isolates, respectively, **(C, E)** PCA-associated loading plots of rice cultivars and *R. solani* isolates, respectively.

All nine isolates were pathogenic and produced typical symptoms of sheath blight disease on 11 different Egyptian rice genotypes under greenhouse conditions. The percentage of relative lesion height ranged from 10.93% (isolate SHBP4 on Egyptian Yasmine) to 68.70% (Isolate SHBP9 on Sakha 104) (**Figure 3A**). Moreover, two-way Hierarchical Cluster Analysis (HCA) and its associated heatmap were performed using the individual response (RLH) of each genotype to different *R*. *solani* isolates (**Figure 3A**). Interestingly, the HCA-associated dendrogram among rice genotypes showed that all tested genotypes were clustered into three distinct clusters. Cluster “I” included three resistant genotypes (Egyptian Hybrid 1, Giza 182, and Egyptian Yasmine), Cluster “II” included four moderately resistant genotypes (Giza 177, Giza 178, Giza 183, and Giza 181), whereas Cluster “II” included four susceptible genotypes (Sakha 101, Sakha 104, Sakha 108, and Sakha Super-300) (**Figure 3A**). It is worth noting that while Egyptian Yasmine showed the highest resistance to all isolates, Sakha 104 had almost the lowest resistance to all isolates. On the other hand, the HCA-associated dendrogram among *R. solani* isolates clustered both SHBP8 and SHBP9 separately from other isolates. *R. solani* AG1 IA isolate SHBP9 was the most aggressive isolate and caused the highest relative lesion height percentage on Sakha 104 (68.70 ± 1.22%), Sakha 101 (64.32 ± 1.14%), Sakha Super 300 (62.53 ± 1.04%), and Sakha-108 (59.40 ± 0.95%) (**Figure 3A**). On the other hand, Egyptian Yasmine was the lowest susceptible rice genotype and recorded the lowest relative lesion height percentage when infected with isolates SHPB4 (10.93 ± 0.51%), SHPB8 (11.02 ± 0.51%), SHPB1 (11.14 ± 0.42%), and SHPB5 (11.30 ± 0.72%) (**Figure 3A**).

Likewise, principal component analysis (PCA) revealed the differences between rice genotypes as well as *R. solani* isolates (**Figures 3B–E**). Briefly, The PCA-associated scatter plot showed a clear separation among all studied rice genotypes concerning the total variation of PC1 (91.47%) and PC2 (2.85%) (**Figures 3A, B**). in agreement with HCA, Egyptian Hybrid 1, Giza 182, and Egyptian Yasmine were clustered together at the left side of the scatter plot and separately from other genotypes. Moderately resistant genotypes (Giza 177, Giza 178, Giza 183, and Giza 181) were grouped in the center of the scatter plot, whereas susceptible rice genotypes (Sakha 101, Sakha 104, Sakha 108, and Sakha Super-300) were grouped into the right side of the scatter plot (**Figure 3B**). Moreover, the PCA-associated loading plot showed that all *R. solani* isolates were positively correlated with the susceptible rice genotypes (**Figure 3C**).

Similarly, the PCA-associated scatter plot of *R. solani* isolates showed a clear separation among all isolates concerning the total variation of PC1 (70.59%) and PC2 (17.48%) (**Figures 3D, E**). The most aggressive isolate, *R. solani* isolate AG1 IA SHBP9, and the lowest aggressive isolate, *R. solani* isolate AG1 IA SHBP8 were grouped far away from each other (**Figure 3D**). Furthermore, the PCA-associated loading plot showed that all rice genotypes were positively correlated with the virulence of *R. solani* AG1 IA isolate SHBP9 (**Figure 3E**).

### 3.6 Assessment of *R. solani* AG1 IA (isolate SHBP9) host range at greenhouse condition

**Figure 4:**
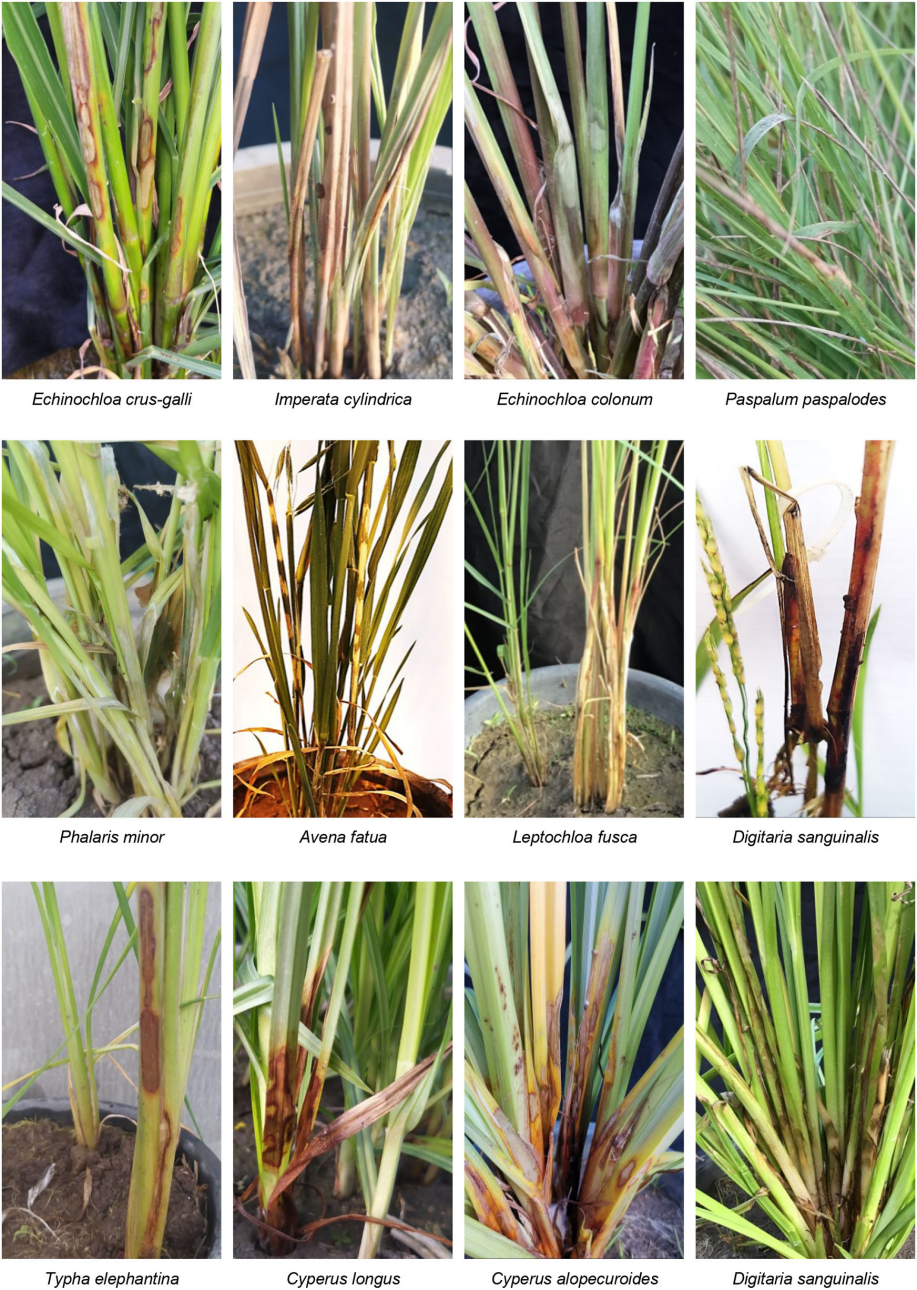
Symptoms’ development of sheath blight disease induced by *Rhizoctonia solani* AG1 IA - isolate SHBP9 on 12 common rice-associated weeds from Poaceae under greenhouse conditions.

**Figure 5:**
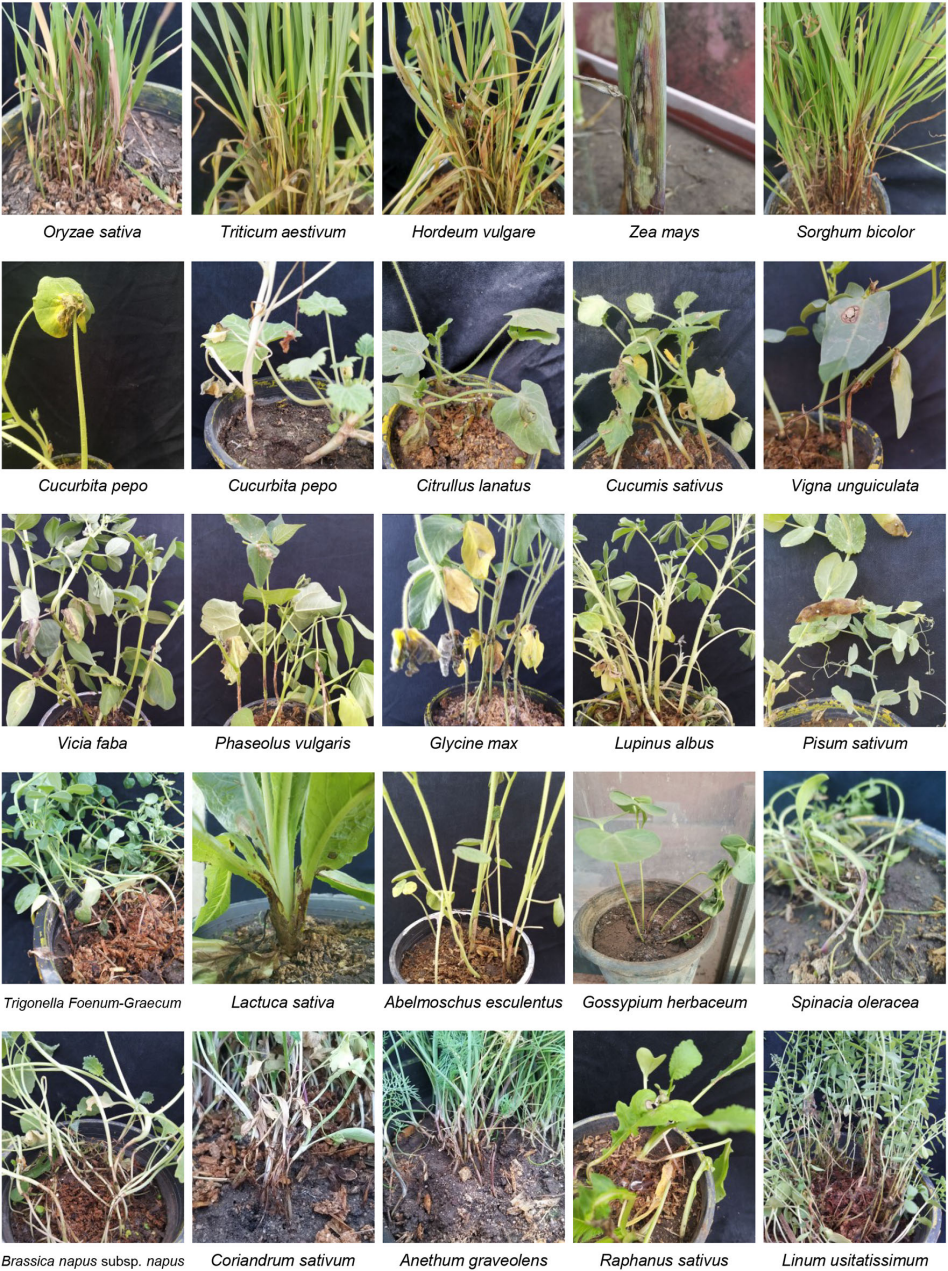
Symptoms’ development of sheath blight disease and similar symptoms induced by *Rhizoctonia solani* AG1 IA - isolate SHBP9 on economic crops from different families under greenhouse conditions.

The virulence of *R*. *solani* isolate AG1 IA SHBP9 was tested against 12 common rice-associated weeds (**Table 1**), and 31 economic crops (**Table 2**) belonging to 12 families (3 Monocotyledons and 9 Dicotyledons) to assess the host range of *R. solani* AG1 IA. All the Monocotyledon plants, 13 of Poaceae, 3 of Cyperaceae, and 1 of Typeaceae, were susceptible to *R. solani* AG1 IA isolate SHBP9 (**Figures 4**, **5**). Meanwhile, 6 plant species belonging to Dicotyledons were resistant to the SHBP9 including chickpea (*Cicer arietinum*) from Fabaceae, Rocket (*Eruca sativa*) from Brassicaceae, and the four studied crops from Solanaceae (potato, tomato, eggplant, and pepper) (**Table 2**). In addition, the remaining 20 Dicotyledons plant species: 3 of Cucurbitaceae, 2 of Malvaceae, 1 of Linaceae, 8 of Fabaceae, 2 of Brassicaceae, 1 of Amaranthaceae, 2 of Apiaceae, and 1 of Asteraceae, were susceptible to *R. solani* isolate AG1 IA isolate SHBP9 (**Figure 5**; **Table 2**).

### 3.7 Evaluation of rice genotypes under artificial inoculation with *R. solani* AG1 IA (isolate SHBP9) under greenhouse conditions

**Figure 6:**
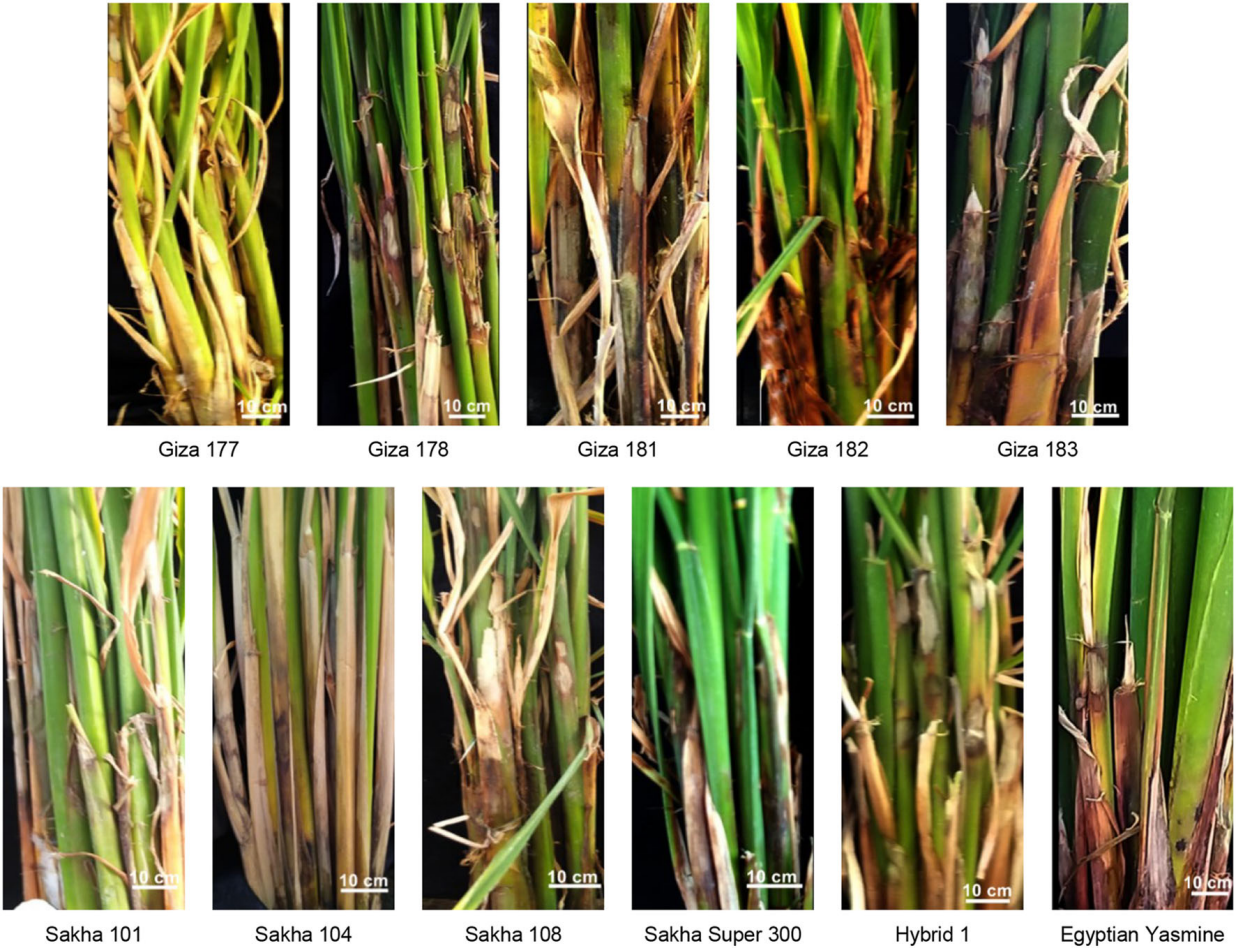
Symptoms’ development of sheath blight disease induced by *Rhizoctonia solani* AG1 IA - isolate SHBP9 on eleven rice genotypes under greenhouse conditions.

**Figure 7:**
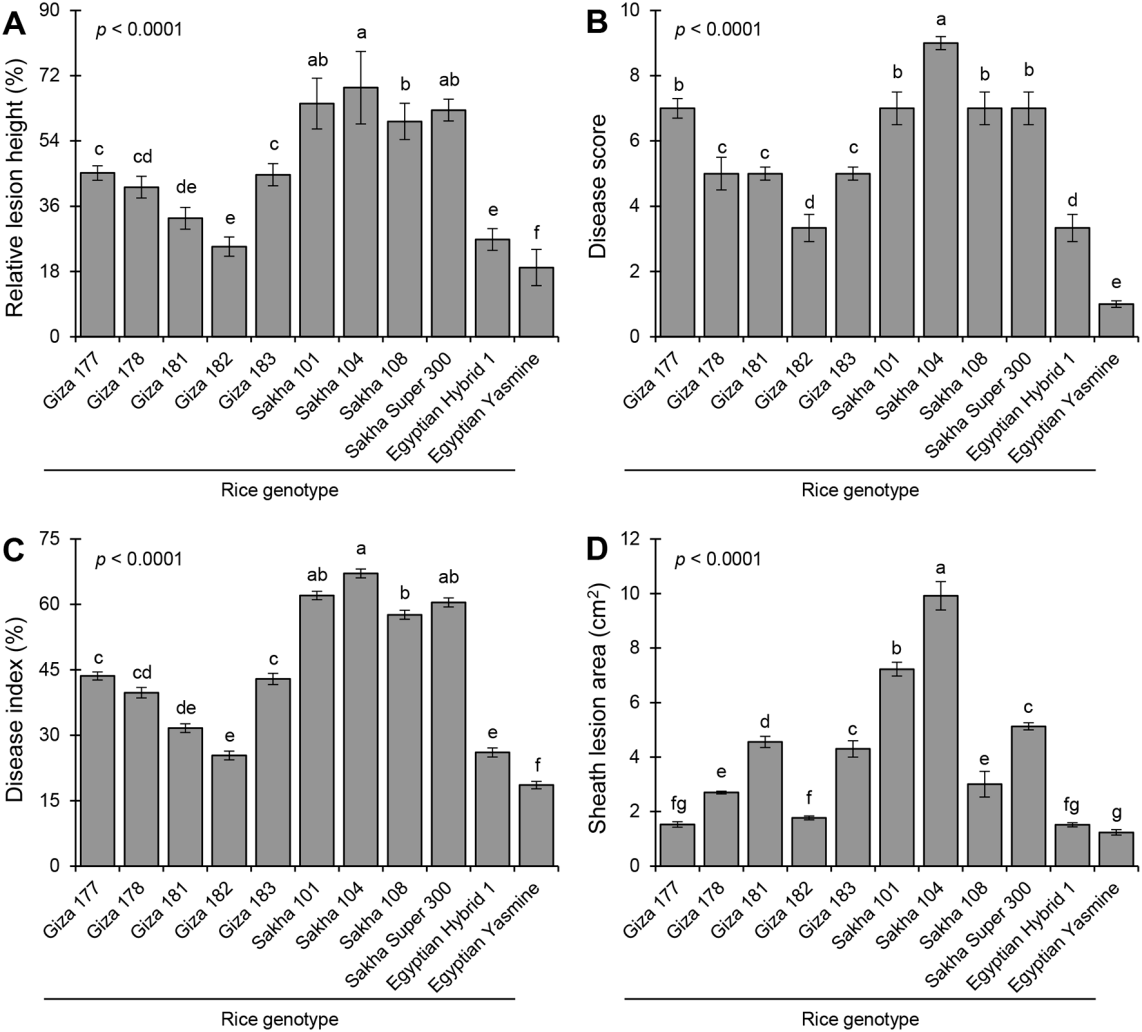
Sheath blight disease assessment of 11 rice genotypes at 30 days post inoculation (dpi) with *Rhizoctonia solani* under greenhouse conditions. **(A)** Relative lesion height (%), **(B)** Disease score, **(C)** percent disease index, and **(D)** sheath lesion area (cm^2^). Bars represent the average of three biological replicates (n = 3), whereas whiskers represent the standard deviation (means ± SD). Different letters signify statistically significant differences among rice cultivars using the Tukey HSD test (p< 0.05).

Eleven rice genotypes (five Japonica, three Indica-Japonica, and three Indica) were used to evaluate their resistance against the sheath blight disease caused by *R. solani* AG1 IA (isolate SHBP 9). Greenhouse findings showed that the typical symptoms of sheath blight disease were observed on the eleven tested rice genotypes at least 30 dpi, (**Figure 6**). Rice genotypes differed in their susceptibility to sheath blight (**Figures 6**, **7**). Sakha 104 recorded the highest RLH (69.63%; **Figure 7A**), disease score (9; **Figure 7B**), disease index (67.31%; **Figure 7C**), and sheath lesion area (9.75 cm^2^; **Figure 7D**), followed by Sakha 101, Sakha super 300, Sakha 108 and Giza 177 in terms of RLH (64.22, 62.52, 59.90 and 45.16%), disease score (7), disease index (62.08, 60.44, 57.93 and 43.65%), and lesion sheath area (7.18, 5.13, 2.96 and 1.53 cm^2^), respectively. Meanwhile, the lowest disease traits were observed by indica type and indica japonica; particularly Egyptian Yasmine with RLH (19.10%), disease score (1), disease index (18.4%), and Lesion sheath area (1.24 cm), followed by Egyptian hybrid 1 and Giza 182 RLH (26.99 and 28.23%), disease score (3), disease index (26.09 and 26.95%) and Lesion sheath area (1.60 and 1.77 cm, respectively). In addition, indica type, i.e., Giza 181, Giza 182, Giza 178, and Giza 183, recorded moderate disease traits ranging from 32- 44.75% for RLH and 5 for disease score (**Figures 7A, B**).

### 3.8 Morphological traits of rice genotypes inoculated with *R. solani* AG1 IA (isolate SHBP9) under greenhouse conditions

**Figure 8:**
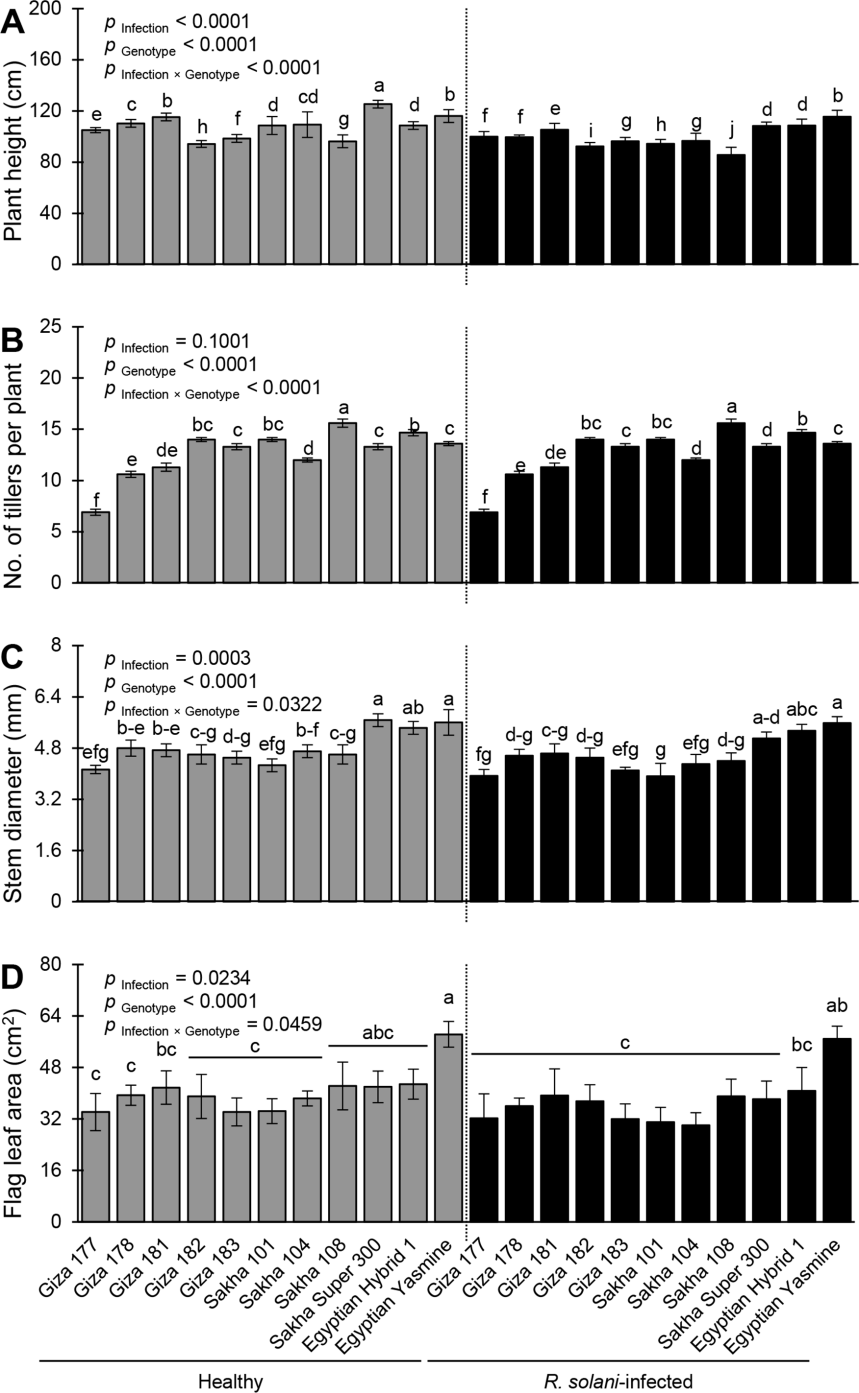
Performance and morphological traits of 11 rice genotypes with or without the infection with ***Rhizoctonia*** *solani* AG1 IA – isolate SHBP9 under greenhouse conditions. **(A)** plant height (cm), **(B)** number of tillers per plant, **(C)** stem diameter (mm), and **(D)** Flag leaf area (cm^2^). Bars represent the average of three biological replicates (n = 3), whereas whiskers represent the standard deviation (means ± SD). Different letters signify statistically significant differences among cultivars using the Tukey HSD test (p _Infection × Genotype_< 0.05).

The morphological traits of healthy and *R. solani*-infected rice genotypes differed significantly (**Figure 8**). Infection with *R. solani* significantly altered plant height (*p*_Infection_< 0.0001; **Figure 8A**), stem diameter (*p*_Infection_ = 0.0003; **Figure 8C**), and flag leaf area (*p*_Infection_ = 0.0234; **Figure 8D**), but not the number of tillers per plant (*p*_Infection_ = 0.1001; **Figure 8B**). Mock-inoculated (healthy) Sakha Super 300 had the plant height (125.3 ± 3.00 cm), whereas *R. solani*-infected Sakha 108 showed the lowest plant height (85.60 ± 6.00 cm) (**Figure 8A**). Likewise, healthy Giza 182 recorded the highest number of tillers per plant (15.60 ± 0.40) which was similar to the *R. solani-*infected Sakha 108 (15.60 ± 0.40) with no significant differences between them (**Figure 8B**). However, Giza 177, either infected or not, had the lowest number of tillers per plant (6.90 ± 0.30). Meanwhile, healthy Sakha Super 300, healthy Egyptian Yasmine, and *R. solani*-infected Egyptian Yasmine had the highest stem diameter (5.67 ± 0.20 5.60 ± 0.40, and 5.58 ± 0.20 mm, respectively) without significant differences between them. Nevertheless, infected Giza 177 and Sakha 101 recorded the thinnest stem diameter (3.93 ± 0.20 and 3.92 ± 0.40 mm, respectively) without significant differences between them (**Figure 8C**). Finally, the flag leaf area did not differ significantly among most of the rice genotypes, either infected or not. However, healthy Egyptian Yasmine had the highest flag leaf area (58.30 ± 4.0 cm^2^), whereas *R. solani*-infected Sakha 104 had the lowest flag leaf area (30.04 ± 3.89 cm^2^) (**Figure 8D**).

### 3.9 Yield components of rice genotypes inoculated with *R. solani* AG1 IA (isolate SHBP9) under greenhouse conditions

**Figure 9:**
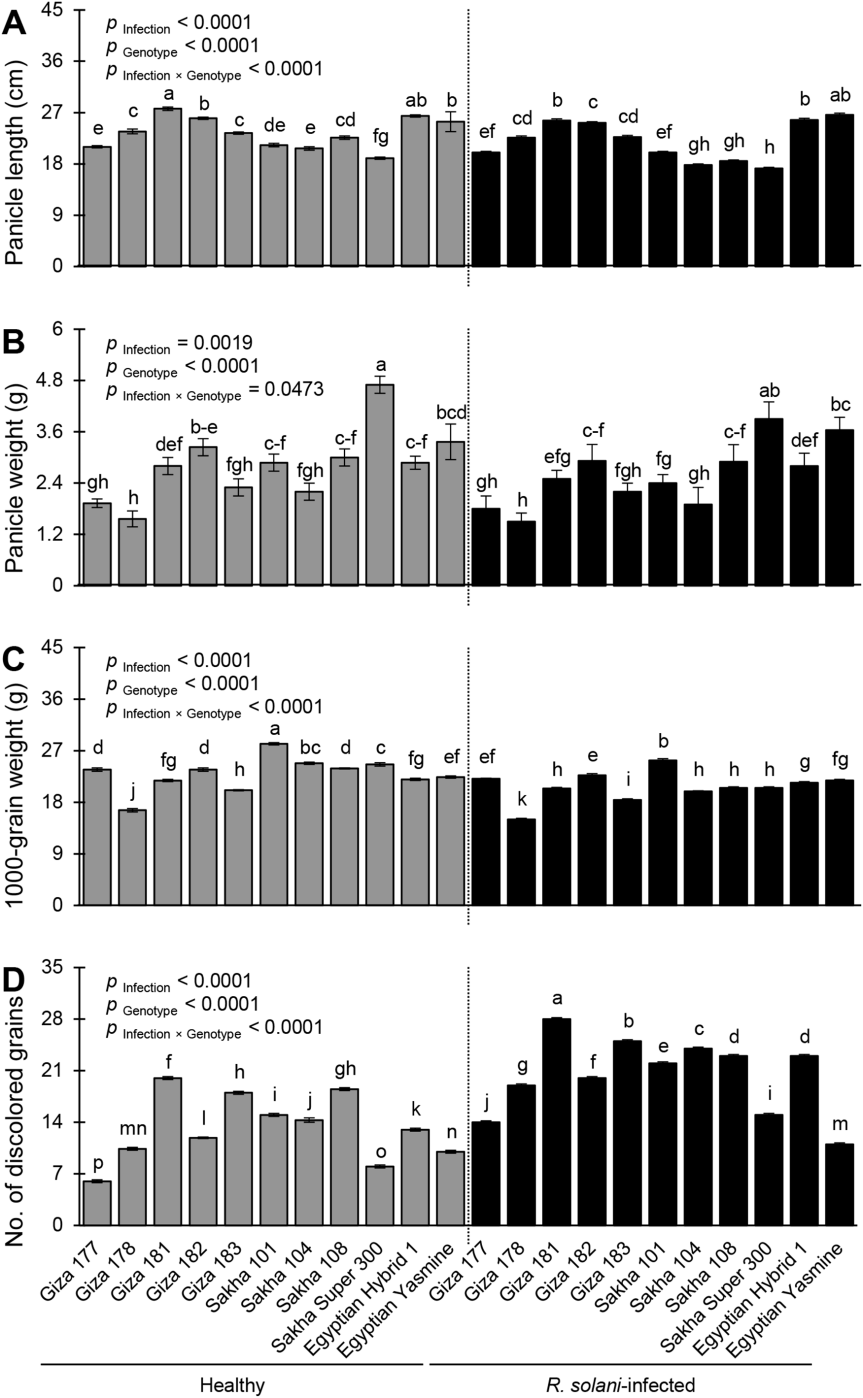
Yield components of 11 rice genotypes with or without the infection with *Rhizoctonia solani* AG1 IA – isolate SHBP9 under greenhouse conditions. **(A)** Panicle length (cm), **(B)** Panicle weight (g), **(C)** 1000-grain weight (g), and **(D)** number of discolored grains. Bars represent the average of three biological replicates (n = 3), whereas whiskers represent the standard deviation (means ± SD). Different letters signify statistically significant differences among cultivars using the Tukey HSD test (p _Infection × Genotype_< 0.05).

Infection with *R. solani* significantly altered all studied yield components including panicle length (*p*_Infection_< 0.0001; **Figure 9A**), panicle weight (*p*_Infection_ = 0.0019; **Figure 9B**), 1000-grain weight (*p*_Infection_< 0.0001; **Figure 9C**), and number of discolored grains (*p*_Infection_< 0.0001; **Figure 9D**). Mock-inoculated Giza 181 recorded the highest panicle length (27.67 ± 0.30 cm), whereas infected Sakha Super 300 had the lowest panicle length (17.20 ± 0.20cm) (*p*_Infection × Genotype_< 0.0001; **Figure 9A**). Likewise, the highest performance of panicle weight was recorded by the healthy Sakha Super 300 (4.70 ± 0.20 g) while the lowest performance of panicle weight was recorded by healthy Giza 178 (1.56 ± 0.20 g) and *R. solani*-infected Giza 178 (1.50 ± 0.20 cm) with no significant differences between them (*p*_Infection × Genotype_ = 0.0473; **Figure 9B**). In terms of 1000-grain weight, non-infected Sakha 101 had the highest 1000-grain weight (28.2 ± 0.20 g) whereas infected Giza 178 had the lowest value (15.00 ± 0.20 g) (*p*_Infection × Genotype_< 0.0001; **Figure 9C**). Finally, *R. solani*-infected Giza 181 recorded the highest number of discolored grains (28.00 ± 0.20) whereas Giza 177 had the lowest number (6.00 ± 0.20) (*p*_Infection × Genotype_< 0.0001; **Figure 9D**).

### 3.10 Behavior and development of the fungal mycelium on the sheath surface of rice genotypes inoculated with *R. solani* AG1 IA (isolate SHBP9) under greenhouse conditions

**Figure 10:**
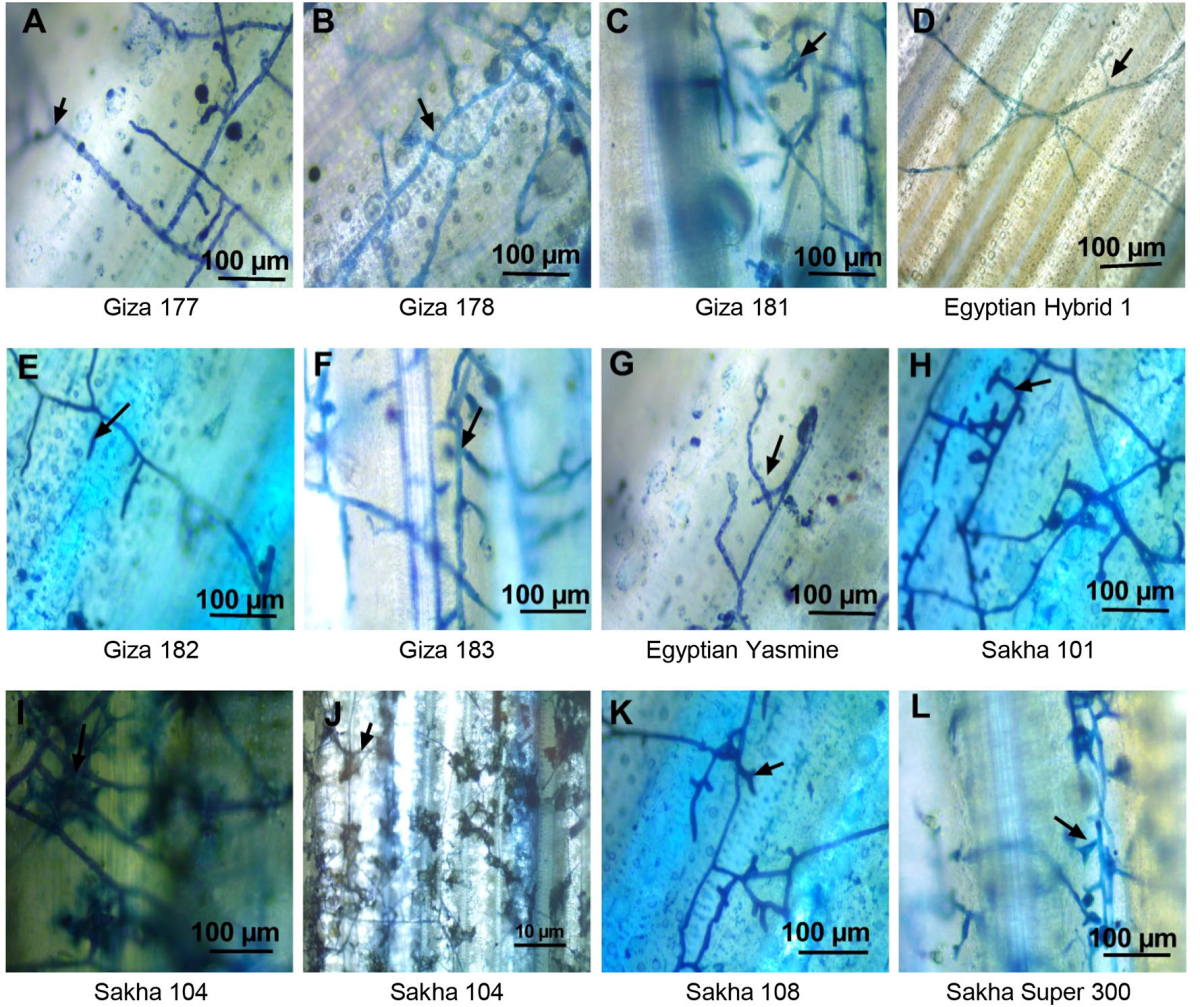
Light microscopy analysis of the behavior and development of the mycelium of *Rhizoctonia solani* AG1 IA – isolate SHBP9 on the surface of infected sheaths of 11 rice genotypes with different degrees of susceptibility. **(A)** Typical mycelium of *R. solani* on the sheath surface of Giza 177 rice cultivar with flat mycelium growth and distribution with more branches, **(B)** Giza 178 cv increases the mycelium growth in the sheath surface and clamped with trichome, **(C)** Giza 181 more mycelium branching and formed to mycelium cushion, **(D)** Egyptian hybrid 1 only mycelium grows on the surface without branching, **(E)** Giza 182 only mycelium grows on the surface without branching, **(F)** Giza 183 more mycelium branching, **(G)** Egyptian Yasmine with aerial mycelium growth no branch, **(H)** Sakha 101 branching mycelium penetrated the stem and branched inside, then the mycelium shrank, **(I, J)** Sakha 104 converted to mycelium cushion and formed sclerotia, **(K)** Sakha 108 branching mycelium penetrated the stem and branched inside, then the mycelium shrank, **(L)** Sakha super 300 branching mycelium penetrated the stem and branched inside, then the mycelium shrank. Arrows indicate the mycelium branching.

The behavior of mycelium growth on different genotypes varies due to the genetic background of each genotype (**Figures 10A–L**). Only growing mycelium was noticed on Egyptian hybrid 1, Giza 182, and Egyptian Yasmine (**Figures 10D, E, G**). The growing mycelium started to branch outside the sheath, as in Giza 177 (**Figure 10A**). The branching mycelium penetrated the stem and branched inside it as in Giza 178 and Giza 183, (**Figures 10B, F**), then the mycelium shrank as in Giza 181 and Sakha 108 and Super 300 (**Figures 10C, K, L** respectively), and converted to mycelium cushion as in Sakha 101 (**Figure 10H**), finally formed sclerotia as in Sakha 104 (**Figure 10I**). So, the fungus can invade the sheath of Sakha 104 faster than the other genotypes. It was observed that there was more branching and growth density of the fungus on the Japanese types, especially the Sakha 104, where there was a significant development in the growth of the mycelium and its transformation into a cushion, as well as the formation of sclerotia on the sheath’s surface (**Figure 10J**).

### 3.11 SSR marker associated with morphological traits and sheath blight resistance

**Figure 11:**
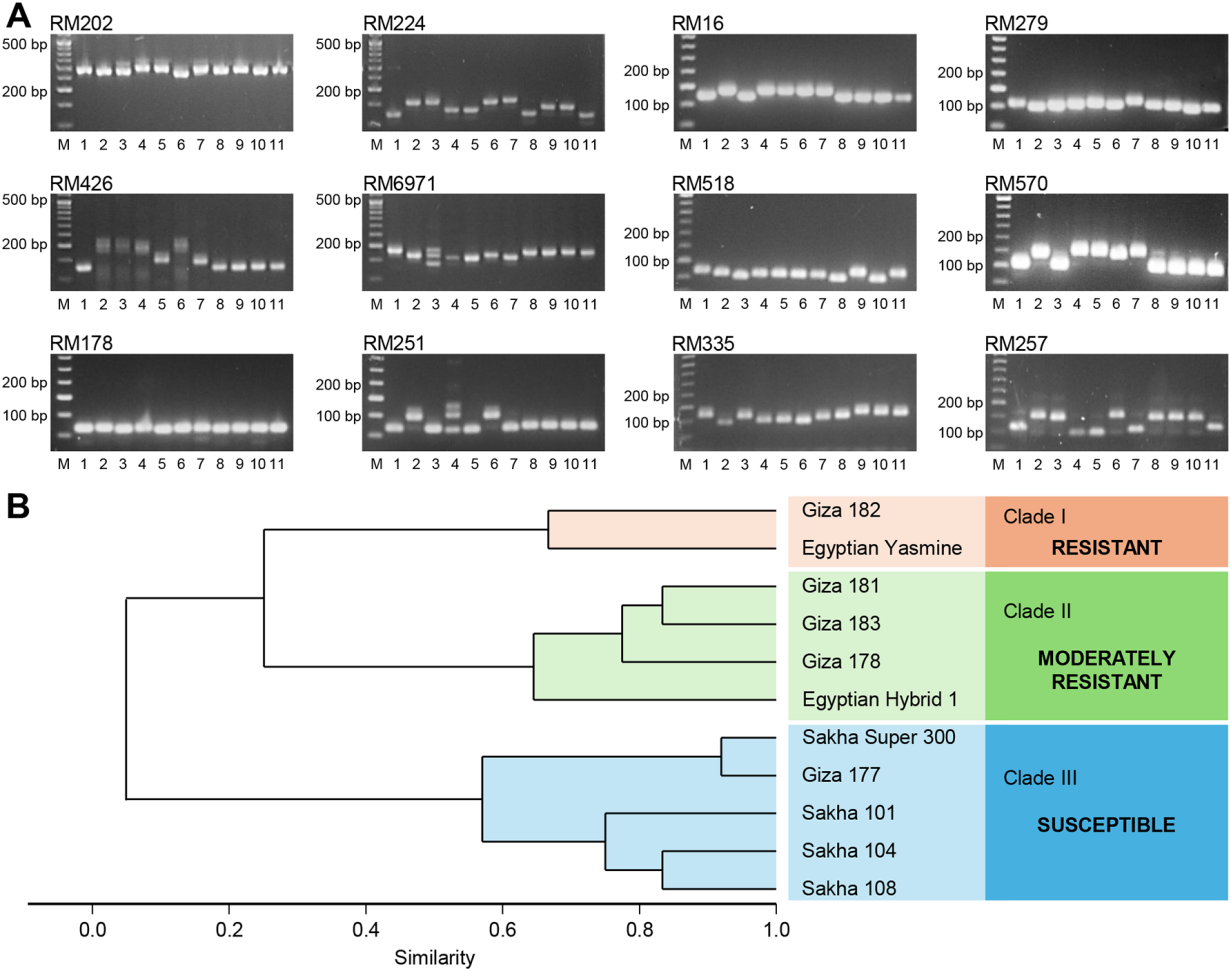
Diversity Analysis of 11 rice genotypes with different degrees of susceptibility based on 12 SSR Markers. **(A)** Representative gel images and banding patterns of 12 SSR markers (RM202, RM224, RM16, RM279, RM426, RM6971, RM518, RM570, RM178, RM251, RM335, and RM257) linked to sheath blight resistance in rice. Lane M represents the standard DNA ladder, whereas lanes 1–11 represent rice genotypes (Giza177, Giza178, Egyptian Hybrid 1, Giza181, Giza182, Giza183, Egyptian Yasmine, Sakha101, Sakha104, Sakha108, and Sakha Super 300, respectively). **(B)** Dendrogram derived from UPGMA cluster analysis of 11 rice genotypes.

Out of the 12 tested SSR markers (**Figure 11A**), 11 SSR markers were polymorphic, and among them, only three markers differentiated between the resistance and susceptible genotypes. Furthermore, these markers, RM202, RM426, and RM6971, showed a specific band (~300, ~144, and ~152bp), respectively, for the resistance genotypes (Egyptian Yasmine and Giza 182) (**Figure 11A**). The SSR—based phylogenetic tree was divided into three main clades (**Figure 11B**). Clade I contained only two resistant genotypes (Giza 182 and Egyptian Yasmine), Clade II contained four moderately resistant genotypes (Giza 181, Giza 183, Giza 178, Egyptian Hybrid 1), and Clade III contained five susceptible genotypes (Sakha Super 300, Giza 177, Sakha 101, Sakha 104, Sakha 108). Finally, these three SSR markers, RM202, RM426, and RM6971, could be used in a breeding program for sheath blight resistance. Collectively, these findings suggest the high specificity of RM202, RM426, and RM6971 markers, as well as their potential utilization in breeding programs since they uniquely differentiate between resistant and susceptible rice genotypes.

## 4 Discussion

*Rhizoctonia solani* is a common fungus found in soil and infects a large number of families (Chahal et al., 2003). Given the importance of this fungus, its pathogenic ability to infect rice plants was recently discovered in Egypt, causing sheath blight disease, especially the strain belonging to the AG1 IA group (Hassan, 2021), and rice worldwide (Wamishe et al., 2007). Isolates differ in their morphological and pathogenic characteristics depending on geographical locations. It was found that the nine isolates of *R. solani* AG1 IA were different in morphological characteristics, their ability to produce some cell wall degrading enzymes, and their pathogenic behavior on rice plants. Many researchers (Moni et al., 2016; Mughal et al., 2017) agree that *R. solani* isolates differ in their morphological and pathological characteristics. This difference may be due to the occurrence of anastomosis compatibility (Mughal et al., 2017) or parasexual cycle (Tinline and MacNeill, 1969; Qu et al., 2008) between *Rhizoctonia* isolates, which leads to the production of new, different strains.

Many pathogenic fungi produce enzymes that degrade the cell wall (Shieh et al., 1997; Have et al., 1998). It was found that *R. solani* can produce these enzymes. The high or low ability to produce these enzymes is due to the virulence of the isolates (Mondal et al., 2013). The fungi benefit from the substances resulting from the process of cell wall decomposition to provide a source of food for the fungus and also facilitate the process of penetration and spread within the host cells (De Lorenzo et al., 1997). Our results showed that *R. solani* AG1 IA isolate SHBP9 excelled in producing the enzymes associated with cell wall degradation and its pathogenic ability to cause infection in rice plants through artificial inoculation under greenhouse conditions. The appearance of symptoms and their severity were positively associated with an increase in the amount of cell wall-degrading enzymes in the susceptible rice cultivar under conditions of infection with *R. solani* (Hassan, 2021).

The necrotrophic fungus *R. solani* is soilborne and is one of the fungi that can grow on the appropriate host to cause sheath blight of rice and cause losses (Senapati et al., 2022). It can grow on some other hosts as a parasite and also cause different symptoms such as root rot, crown, damping off, and black peeling, but it does not cause losses (Nagaraj et al., 2017; Shekhawat et al., 2023). Species of *R. solani* are classified into multinucleate and binucleate isolates based on the number of nuclei inside each cell and each of these groups is divided into anastomosis groups (AGs) based on the ability of hyphae to fuse (Lee et al., 2021). To date, 21 AGs (named AG-A to AG-U) were reported within the binucleate group, whereas only 13 AGs (named AG-1 to AG-13) were reported within the multinucleate group (Muzhinji et al., 2015; Muzhinji and Lekota, 2024). In the current study, all nine tested *R. solani* isolates were classified within the multinucleate group AG1-IA. Moreover, the most aggressive isolate *R*. *solani* AG1 IA isolate SHBP9 was virulent on 12 common rice-associated weeds, and 25 economic crops belonging to 12 families, however, it did not show any symptoms on chickpea from Fabaceae, rocket from Brassicaceae, and the four crops from Solanaceae including potato, tomato, eggplant, and pepper. It was reported previously that seven AGs were associated with wet root rot in chickpea including AG1, AG2-2, AG2-2LP, AG2-3, AG3, AG4, and AG5 (Ganeshamoorthi and Dubey, 2013). Likewise, rocket (*Eruca sativa*) seedlings were susceptible to *R. solani* – AG-4 and exhibited strong damping-off symptoms (Pane et al., 2020). Nevertheless, in the current study, *R*. *solani* AG1 IA isolate SHBP9 did not show any significant symptoms either on chickpea or rocket plants under greenhouse conditions.

Likewise, several previous studies experimentally reported the susceptibility of potato and other solanaceous plants to *R. solani* – AG-3 (Woodhall et al., 2007; Tsror, 2010; Yarmeeva et al., 2021; Naqvi et al., 2024), particularly AG-3 PT (Zrenner et al., 2020; Hejazi et al., 2022; Iradukunda et al., 2022; Monazzah et al., 2022). The most updated AG distribution in potato worldwide confirms the association of some other AGs rather than only AG-3 PT such as AG2-1 (Woodhall et al., 2007), AG-5, and AG-K (Yarmeeva et al., 2021), AG-2, AG-4 (HGI and HGII), AG-6, AG-7, and AG-9 (López-Corrales et al., 2024) which markedly reflects the population dynamics of the pathogen. Recently, several *R. solani*-AG-1 (AG-1 IA, AG-1 IB, and AG-1 IC) were reported to be associated with potato diseases in Northern Sinaloa, Mexico (López-Corrales et al., 2024). However, the aggressive Egyptian *R*. *solani* AG1 IA isolate SHBP9 (isolated from rice) did not show any symptoms on potato plants under greenhouse conditions. These findings suggest potential differences in host specificity and pathogenicity among *R. solani* isolates, even within the same anastomosis group (AG-1). Collectively, these findings highlight the complexity of host-pathogen interactions within *R. solani*.

Sheath blight is a disease that is widespread in most rice crops in the world. Due to the limited existing sources of resistance to this disease, we always resort to using alternative treatments to stop the spread of the disease and limit its losses (Molla et al., 2020). However, varieties that have partial resistance to this disease are considered among the best environmentally friendly and safe methods (Hassan, 2021). Therefore, searching for sources of resistance in Egyptian cultivated varieties is one of the important means of resisting the disease. The economic losses for the sheath blight disease vary among genotypes; thus, the identification of resistance sources is very important. Rice cultivars (Indica type) showed resistance to sheath blight under artificial infection conditions with the *R. solani*, with less loss in 1000-grain weight compared to Japonica-type varieties, which were more susceptible to the fungus and also the greatest loss in weight per thousand grains, under the same conditions.

Map populations produced from indica or japonica rice have been used to identify several quantitative trait loci (QTL) for sheath blight resistance. Different rice cultivars exhibiting partial resistance have mapped numerous QTLs, including Tetep, Teqing, jasmine, and additional germplasm lines (Bal et al., 2020). It has been found that cultivars from the indica subspecies are more resistant to sheath blight than cultivars from the japonica subspecies (Jia et al., 2012). The study measured the morphological traits of the infection and non-infected varieties, and the loss was determined. It was found that the Indica type (Egyptian Yasmine and Giza 182) is the least affected by the infection. The lesser susceptibility to infection may be due to the difference in morphological traits in the Indica-type cultivars from the Japanese ones. It was found that the Egyptian Yasmine variety has a higher height than other varieties, as well as the area of the flag leaf, so the fungus may take longer to reach the panicle and cause loss compared to the Japanese types. Hossain et al. (2016) found that morphological traits of rice genotypes were associated with resistance to sheath blight disease. The morphological characteristics of the cultivars may be positively related to the environmental conditions (Groth and Nowick, 1992) that lead to reducing the severity of the infection. Plant growth factors such as plant height, and flag leaf area indirectly affect rice sheath blight resistance (Zou et al., 2000; Pinson et al., 2005). It was found that the area of the flag leaf in the Egyptian Yasmine was large compared to other varieties. Perhaps the larger leaf width increases the area exposed to solar radiation and thus reduces the disease.

Rice sheath blight is a disease controlled by polygenes and is a quantitative trait linked to morphological traits (Hossain et al., 2016). Twelve SSR markers divided rice genotypes into three groups: susceptible genotypes (japonica type), moderately resistant genotypes (indica/japonica and indica type), and resistance genotypes (indica type). Many researchers (Willocquet et al., 2012; Wen et al., 2015) have agreed that Indica-type genotypes are more resistant than japonica types. Although most tested SSR markers in this study showed polymorphism among tested genotypes without a clear indication of resistance among rice genotypes, three markers RM202, RM426, and RM6971 demonstrated a specific band (~300, ~144, and ~152bp, respectively) only in resistant genotypes like Egyptian Yasmine and Giza 182. The high specificity of these three markers suggests their potential as a reliable genetic source for identifying resistance traits at the molecular level, vital for selecting disease-resistant rice genotypes. It is well known that traditional breeding for disease resistance in cereal crops, particularly rice, is time-consuming and significantly affected by environmental variations. We believe that RM202, RM426, and RM6971 could be used as accurate molecular indicators for sheath blight resistance during the marker-assisted selection (MAS) breeding programs to save time and resources. Briefly, screening rice seedlings for these markers at early stages might help breeders identify resistant materials without waiting for field-based disease assessments which will accelerate the development of resistant rice varieties and augment the reliability of breeding outcomes.

## 5 Conclusion

Sheath blight, caused by *R. solani*, is a serious emerging disease of rice in Egypt and worldwide. Although all characterized *R. solani* isolates fall within the anastomosis group anastomosis group (AG) AG-1 IA, virulence variability was noticed due to the variation in their ability to produce CWDEs, as well as geographical distribution. *R. solani* isolate AG1 IA SHBP9 (PP445325.1) was the most aggressive isolate and was able to infect 12 common rice-associated weeds from Poaceae and several hosts from different families, except some hosts of the family Solanaceae and Fabaceae. Indica rice genotypes (such as Giza 182 and Egyptian Yasmine) and Indica/Japonica rice genotypes (such as Giza 178 and Giza 183) were more resistant to *R. solani* than Japonica rice genotypes (Sakha 101, Sakha 104, and Sakha 108). Accordingly, we encourage the adoption and cultivation of resistant genotypes such as Egyptian Hybrid 1, Giza 182, and Egyptian Yasmine, rather than the susceptible genotypes such as Sakha 101, 104, 108, and Super-300 under Egyptian conditions. However, further studies are required to confirm the resistance behavior of commercial rice cultivars over the years, particularly the moderately resistant genotypes such as Giza 177, 178, 181, and 183 which are threatened to be more susceptible to *R. solani* under the current climate change and the invasion of new isolates. While we acknowledge the limited number of isolates and genotypes in the current study, we believe that future studies with expanded sample sizes could provide additional insights into this emerging disease.

## Data Availability

The original contributions presented in the study are included in the article/Supplementary Material. Further inquiries can be directed to the corresponding authors.
